# Virtual tissue expression analysis

**DOI:** 10.1093/bioinformatics/btae709

**Published:** 2024-11-26

**Authors:** Jakob Simeth, Paul Hüttl, Marian Schön, Zahra Nozari, Michael Huttner, Tobias Schmidt, Michael Altenbuchinger, Rainer Spang

**Affiliations:** Institute for Statistical Bioinformatics, Faculty of Informatics and Data Science, University of Regensburg, Am Biopark 9, 93053 Regensburg, Germany; NGS and Data Technologies Core, Leibniz Institute for Immunotherapy (LIT), c/o Universitätsklinikum Regensburg, Franz-Josef-Strauss Allee 11, 93053 Regensburg, Germany; Institute for Statistical Bioinformatics, Faculty of Informatics and Data Science, University of Regensburg, Am Biopark 9, 93053 Regensburg, Germany; Institute for Statistical Bioinformatics, Faculty of Informatics and Data Science, University of Regensburg, Am Biopark 9, 93053 Regensburg, Germany; Institute for Statistical Bioinformatics, Faculty of Informatics and Data Science, University of Regensburg, Am Biopark 9, 93053 Regensburg, Germany; Institute for Statistical Bioinformatics, Faculty of Informatics and Data Science, University of Regensburg, Am Biopark 9, 93053 Regensburg, Germany; Institute for Statistical Bioinformatics, Faculty of Informatics and Data Science, University of Regensburg, Am Biopark 9, 93053 Regensburg, Germany; Department of Medical Bioinformatics, University Medical Center Göttingen, 37077 Göttingen, Germany; Institute for Statistical Bioinformatics, Faculty of Informatics and Data Science, University of Regensburg, Am Biopark 9, 93053 Regensburg, Germany

## Abstract

**Motivation:**

Bulk RNA expression data are widely accessible, whereas single-cell data are relatively scarce in comparison. However, single-cell data offer profound insights into the cellular composition of tissues and cell type-specific gene regulation, both of which remain hidden in bulk expression analysis.

**Results:**

Here, we present tissueResolver, an algorithm designed to extract single-cell information from bulk data, enabling us to attribute expression changes to individual cell types. When validated on simulated data tissueResolver outperforms competing methods. Additionally, our study demonstrates that tissueResolver reveals cell type-specific regulatory distinctions between the activated B-cell-like (ABC) and germinal center B-cell-like (GCB) subtypes of diffuse large B-cell lymphomas (DLBCL).

**Availability and implementation:**

R package available at https://github.com/spang-lab/tissueResolver (archived as 10.5281/zenodo.14160846).

Code for reproducing the results of this article is available at https://github.com/spang-lab/tissueResolver-docs archived as swh:1:dir:faea2d4f0ded30de774b28e028299ddbdd0c4f89).

## 1 Introduction 

Present day single-cell studies describe the biology of tissues in great detail. When comparing two categories of tissues, single-cell data directly associates differentially expressed genes with their specific cell origins. For instance, if a group of mitotic genes shows upregulation at the tissue level in a tumor, single-cell data can distinguish whether these genes are expressed in actively dividing tumor cells or, conversely, in expanding immune cell populations that are combating the tumor. However, scRNA-seq data are expensive to generate, and the number of different patient samples is typically much lower than in bulk RNA-seq studies.

Furthermore, due to the relatively recent development of this technology, clinical follow-up data for patients are scarcely available. In contrast, bulk expression data have been systematically gathered in substantial quantities and are well-annotated from a clinical perspective. These data are readily accessible through sources such as the Cancer Genome Atlas (TCGA) (Weinstein *et al.* 2013) or the International Cancer Genome Consortium (ICGC) (Zhang *et al.* 2019).

The quantification of cell populations from bulk tissue sequencing data, known as tissue deconvolution, is a vibrant area of research (Chen and Wu 2021), and deconvolution tools provided important insights into the cellular composition of tissues (Cheng *et al.* 2020). Their effectiveness stems from their ability to analyze large bulk datasets. More recent devonvolution methods distinguish themselves by learning the distinctive characteristics of the specific cell types of interest from single-cell data (Wang *et al.* 2019, Görtler *et al.* 2020). The single-cell data are used to construct reference profiles for each cell type. While this approach is beneficial for estimating cell type frequencies, it unavoidably simplifies the intricate regulation processes within individual cell populations, which cannot be fully encompassed by a cell type label alone.

Recent methodologies fine-tune the reference profiles to the particular tissue type of interest (Newman *et al.* 2019, Chen *et al.* 2020, Görtler *et al.* 2020, Görtler *et al.* 2023). However, even with these advancements, the within-tissue diversity of cells still remains concealed. The challenge, therefore, lies in bridging the gap between bulk RNA sequencing data from extensive cohorts with comprehensive clinical information and single-cell RNA sequencing, which offers a precise depiction of tissue characteristics.

When we have knowledge of cell type frequencies within the bulk tissue, it becomes feasible to attribute bulk gene expression of specific genes to individual cell types (Shen-Orr *et al.* 2010, Yoshihara *et al.* 2013, Wang *et al.* 2019, Görtler *et al.* 2023). Nonetheless, a strict cell type counting approach fails to encompass the considerably more nuanced, gradual molecular variations that occur within cells of the same type. Cells can undergo activation or reprogramming in numerous different ways, and their continually evolving molecular phenotypes give rise to intricate trajectories in a multidimensional space, which is far more intricate than a mere list of discrete cell types (Frishberg *et al.* 2019).

In groundbreaking work, BayesPrism (Chu *et al.* 2022) departs from the notion of fixed cell types and instead strives for a simultaneous estimation of cellular phenotypes and their frequencies within a tissue. BayesPrism is an exceptionally comprehensive full Bayesian model that quantitatively encompasses a wide array of facets in tissue biology, spanning from cellular composition to gene regulation, and from cell plasticity to regulatory dynamics. Similarly, ISLET (Feng *et al.* 2023) and bMIND (Wang *et al.* 2021) are population-based methods addressing cell-type specific gene expression on the level of sample groups rather than individual samples.

Here, we introduce “tissueResolver,” an alternative, strictly regression-based approach for generating virtual tissues. Unlike standard deconvolution approaches, this method operates without the need for predefined cell type labels. Instead, it autonomously identifies distinct cell populations directly from the scRNA data, exhibiting regulatory differences across various tissues.

We conducted performance tests on tissueResolver using simulations involving bulk data derived from modified single-cell data. In this process, we also explored the boundaries of its applicability. We observed that tissueResolver excels over state of the art cell type-specific expression estimation algorithms when applied to the same scenarios, particularly in terms of its ability to provide higher resolution and specificity when attributing expression changes to specific cell types.

In a case study involving solid tumor diffuse large B-cell lymphomas (DLBCL) samples, tissueResolver provided fresh insights into the subtle expression differences between its activated B-cell-like (ABC) and germinal center B-cell-like (GCB) subtypes. Notably, these differences extended beyond the tumor cells themselves to encompass various elements of the tumor microenvironment. Specifically, we were able to identify regulatory effects within a small cluster of cells exhibiting expression profiles resembling fibroblasts (Steen *et al.* 2021), which would have remained hidden when subsuming all these cells under a pre-assigned cell type label.

## 2 Concept

**Figure 1.**
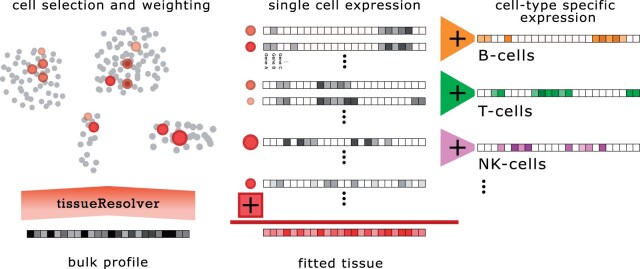
The tissueResolver concept: a bulk expression profile gets reconstructed using the input single-cell library by selecting and weighting the most suitable cells. The fitted tissue is then a fully resolved approximation of the actual bulk whose decomposition can be studied on the cell type-specific level.

The concept of tissueResolver can be seen as analogy to a conductor recreating the sound recording of an orchestral piece by ear. A conductor can recreate the sound heard, the bulk expression profile in our image, by choosing adequate instrumentalists and by giving the right instructions in terms of dynamics, tempo, etc. She or he will select from a large pool of instrumentalists, the single-cell library in our image, just as tissueResolver will only pick the most suitable cells in order to explain the bulk. But apart from this mere selection, the conductor also shapes the dynamics of each instrumentalist contributing to a well-balanced orchestral body. Although this image does not represent the temporal dynamics in cell biology, it illustrates how our algorithm works: it takes a bulk tissue expression profile and a large library of single cells as inputs and then aims to reconstruct the bulk profile as a weighted sum of selected single-cell profiles. These chosen cells can then be analyzed in a similar way as any single-cell dataset: typically, cells are categorized and averaged according to their cell type, resulting in gene expression profiles specific to each cell type. Notably, when substituting one bulk tissue sample with another, while maintaining the identical single-cell library, tissueResolver is likely to select a different subset of single cells. This process leads to the derivation of distinct cell type-specific expression profiles for each bulk sample, see Fig. 1.

## 3 Algorithm

### 3.1 Computation of virtual tissues

We approach the following problem: given a bulk expression profile and a library of single-cell profiles commonly normalized on a linear scale, e.g. CPM or TPM, we aim to determine the optimal subset of cells and their corresponding weights in such a way that the sum of their weighted expressions closely approximates the bulk expression profile in euclidean distance.

Formally, we minimize:

(1) L(β)=||Y−Xβ|| subject to β≥0,

where $Y\in \;R_{+}^{N_{g}\times N_{s}}$ is a matrix of *N_s_* samples, $X\in \;R_{+}^{N_{g}\times N_{c}}$ the library of single-cell profiles, and *N_g_* is the number of genes in the profiles. The parameters that we wish to determine are the entries of the weight matrix $\beta \in \;R_{+}^{N_{c}\times N_{s}}$. We do so using non-negative regression (Slawski and Hein 2013) resulting in a sparse non-negative estimate of *β*. To further stabilize the estimate, we bootstrap the single-cell data *N_k_* times using a random 10% percent of cells and rerun the regression analysis, see also Supplementary Material. This results in a set of *N_k_* different single-cell libraries and corresponding sparse weight estimates that are analyzed separately and in the end results are averaged. When following this approach, we assume that the single-cell library is comprehensive and contains cells of most types and states present in the bulk tissue. We further assume that the bulk expression profile can be effectively reconstructed by selecting the appropriate cells from this extensive collection. However, not all cell types or cell states from the library will be present in every bulk tissue. Therefore, we use a sparse estimate of *β*. The resulting combination of weights *β* and single-cell profiles *X* essentially decomposes the bulk tissue into a selection of single-cell profiles. Consequently, we call this profile composition a “virtual tissue.” The virtual single-cell profiles can be further subjected to downstream analyses in a manner similar to real single-cell data.

Even though tissueResolver is conceptually different from other deconvolution approaches, it could be seen as a maximally fine-grained deconvolution in which each single cell is treated as its own “cell type.” In the library, there may be many cells of the same type, like CD8+ T cells, but they may be in different expression states, such as activated and inactivated. If the algorithm predominantly selects activated T cells in tissue A and inactivated T cells in tissue B, we can observe changes in gene expression that are clearly associated only to T cells. Changes in gene expression were already observable in the bulk profile. However, it became clear only at the virtual tissue level that these genes were differentially expressed within CD8+ T cells. In classical deconvolution approaches, the matrix *X* contains only a few columns, typically one for each included cell type. In this setup, the linear equation system Y−Xβ does not capture full heterogeneity across cells of the same type. However, in tissueResolver, *X* includes many more columns, one for every cell in the library, which necessitates regularization techniques to handle the increased dimensionality.

We chose non-negative regression for learning the weights *β*. This approach not only ensures that the weights remain non-negative, which is crucial for interpretability, but it also leads to a sparse and regularized estimate of *β* (Slawski and Hein 2013) without additional regularization parameters, and avoids that similar cells can cancel each other out. Cells that are not similar to cells present in the bulk will be assigned weights that are very close to zero. In contrast, for redundant cells that contribute much of the expression found in the bulk, a few representative cells will be chosen that will be assigned large weights.

Selecting an appropriate library is important. The number of non-zero weights tends to be higher when there is a significant overlap between the bulk tissue and the single-cell library. This overlap is more likely when bulk and single-cell data are derived from similar tissues. If cell identities are missing in the library, however, certain genes will not be represented well in the virtual tissue, Xβ, and we find large residuals for these particular genes.

Bootstrapping further helps in quantifying such uncertainties. Large residuals of the final model as well as unstable bootstrap results point to problems in the choice of the library. Furthermore, the gene-wise variance across bootstrap samples serves as a valuable quality metric, assessing the extent to which a particular gene is effectively represented in the single-cell dataset. Only genes that are well covered will be interpreted in subsequent analysis.

### 3.2 Cell frequencies and cell type-specific gene expression from virtual tissues

The previous section did not require any cell type labels for the cells in the library. In fact, tissueResolver operates without requiring cell type labels, distinguishing it from other tools like BayesPrism, see also Supplementary section “Simulations.” However, the introduction of cell types can be advantageous for enhancing our comprehension of tissue biology, as it situates virtual tissues within the framework of conventional cell biology. In the following, we assume a set M of *N_L_* distinct cell type labels and denote with $l\in M^{N_{c}}$ the vector labeling every single cell in *X*. Note that there are many complementary ways to label cells, e.g. cell type annotation, cluster membership, or activation and cell cycle states, and for every choice of *l*, the same virtual tissue can be re-analyzed.

For every cell label a∈M, we can compute effective cell frequencies by summing over the respective weights,

(2) $$c_{s}^{a}=\frac{1}{\sum_{i}\beta_{i,s}}\sum_{\overset{i}{l_{i}=a}}\beta_{i,s},$$

which we repeat for every bootstrap sample in order to compute bootstrap averages and errors in the end. This yields the effective frequency $0\leq c_{s}^{a}\leq 1$ of cells of type *a* in sample *s*.

Note that the values of $c_{s}^{a}$ can only be meaningfully interpreted when comparing the frequency of the same cell type across different tissues. Comparing different cell types within the same tissue, on the other hand, is not valid due to variations in the total amount of expressed RNA across cell types.

Estimating cell type-specific gene expression follows a similar approach. To calculate the estimated expression profile of a cell type *a* in sample *s*, we sum the explained expression over all cells of that particular type,

(3) $${\tilde{Y}}_{\cdot ,s}^{a}=\sum_{\overset{i}{l_{i}=a}}X_{\cdot ,i}\beta_{i,s}.$$

Note that the sum over all cell labels recovers the estimated total gene expression, $\tilde{Y}=X\beta =\sum_{a}{\tilde{Y}}^{a}$.

### 3.3 Quality scores and gene selection

It is crucial to qualitatively assess virtual tissues before proceeding downstream. Some genes within the virtual tissue may not align well with the actual bulk tissue due to the absence of certain cell types in the single-cell dataset or due to technological disparities between bulk and single-cell sequencing. Additionally, individual virtual tissues may have failed to fully account for their respective bulk profiles. Once more, this could indicate inadequate single-cell libraries lacking essential cell identities required for accurate representation of the bulk samples. In the following section, we will explore how spurious genes and virtual tissues can be identified and subsequently excluded from further downstream analysis.

On every bootstrap run *k*, we compute for every gene *g* and sample *s* the relative residual,

(4) $$r_{g,s}^{\left(k\right)}=\frac{\left|Y_{g,s}-{\tilde{Y}}_{g,s}^{\left(k\right)}\right|}{Y_{g,s}+{\tilde{Y}}_{g,s}^{\left(k\right)}}\in [0,1],$$

with values close to zero indicating a perfect fit of gene *g* in sample *s*, while a value near one indicates that the explained gene expression substantially deviates from the actual gene expression in the bulk.

By averaging $r_{g,s}^{\left(k\right)}$ over bulk samples and bootstrap runs, we can assess how effectively gene *g* is reconstructed across all virtual tissues. This results in the gene-specific quality score $g_{g}=\frac{1}{N_{s}N_{k}}\sum_{s,k}r_{g,s}^{\left(k\right)}$. In contrast, averaging over all genes of a virtual tissue, $b_{s}=\frac{1}{N_{g}N_{k}}\sum_{g,k}r_{g,s}^{\left(k\right)}$, gives a quality score for individual virtual tissues.

To judge the robustness of the fit, it is also useful to consider mean bootstrap variances of the above quantities, e.g. $v_{g}=\frac{1}{N_{s}}\sum_{s}{\text{var}}_{k}(r_{g,s})$. Very large values (with respect to its mean value) indicate large fluctuations in the gene expression of the individual bootstrap samples that result from a sensitivity to small communities of individual, possibly outlier cells, see also Supplementary section “Quality control of virtual tissues.”

## 4 Simulations

To evaluate the performance of tissueResolver on its main task of detecting differential signals directly on the single-cell level, and to compare it to BayesPrism (Chu *et al.* 2022), the CIBERSORTx HiRes module (Newman *et al.* 2019) which serves also as cell type-specific purification in EcoTyper (Steen *et al.* 2021), ISLET (Feng *et al.* 2023), and bMIND (Wang *et al.* 2021), we introduce artificial cell type-specific gene expression changes to a single-cell DLBCL dataset (Steen *et al.* 2021), see Supplementary Material. (The data of Steen *et al.* (2021) are publicly available under GEO accession numbers GSE182436 and GSE182434.) We only change a subcompartment of cells belonging to one cell type, effectively introducting within cell type herogeneity and thereby altering cell type-specific gene regulation. This setup challenges tissueResolver to identify (i) the modified genes and (ii) to attribute the changes to only the modified cell type.

The data (Steen *et al.* 2021) comprise 28 416 single cells obtained from eight lymphoma patients, with a total of 6639 CD8+ T cells—the cell type we modified. We randomly divided the eight patients into two groups of four each. The cells from the first group constitute a library *X*, while those from the second group were employed to generate artificial bulk profiles.

We randomly selected half of all CD8+ T cells and multiplied the expression of a varying number of randomly selected genes $\left|G_{\text{mod}}\right|$ by different factors ranging from $2^{0.5}\approx 1.41$ to $2^{2}=4$. The modified genes were not exclusively expressed in CD8+ T cells, but only the fraction that originated from CD8+ T cells was modified, for details see Supplementary section “Simulation details.”

From the second set of samples, we created 100 artificial bulk profiles by randomly selecting 500 single cells and summing over their profiles. Out of these 100 artificial bulks, 50 were drawn from the set where CD8+ T cells were modified, forming class I. The remaining 50 samples exclusively included unmodified T cell profiles, constituting class II. By construction, class I and II only exhibited significant differences in gene expression within the CD8+ T-cell compartment.

**Figure 2.**
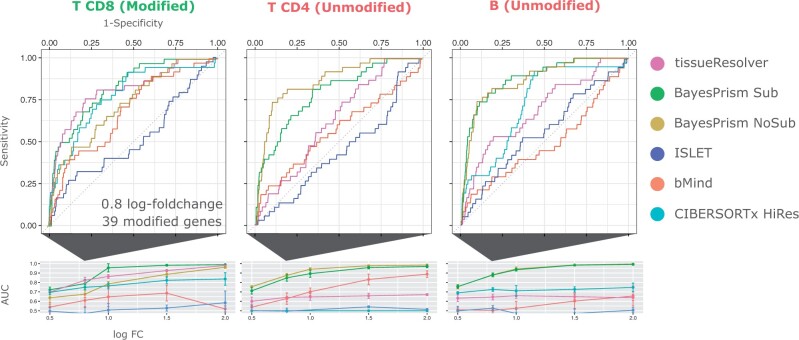
Results of simulations performed for the evaluation of tissueResolver and competing algorithms. *Top row*: ROCs for the modified CD8 T cells and the (unmodified) CD4 T and B computed for finding the 38 modified genes in the top differential expressed genes of the respective cell type for a change in foldchange of $2^{0.8}\approx 1.74$. Two variants of BayesPrism were run: One in which only the annotated cell type was provided (“NoSub”) and one in which each annotated cell type was sub-clustered further (“Sub”), see Supplementary Material for details. *Second row*: Extracted (AUC) values of for finding a modified gene in the top differential genes of the given cell type as a function of the fold change. Values significantly larger than 0.5 mean that the modified genes have been attributed to the corresponding cell type, which we expect only for the modified CD8 T cells, whereas other cell types are expected to give values around 0.5. Note that the ROC curve of CIBERSORTx is not reported here, as in all but one run the predicted CD4 T expression profiles were constant across all bulk samples.

We run all of the above algorithms to estimate cell type-specific gene expression for all cell types. We assessed the performance of all methods in (i) identifying the modified genes and (ii) attributing the differential expression exclusively to CD8+ T cells. Therefore, for each cell type separately, we ranked genes based on differential gene expression and computed receiver operating characteristics (ROC) and areas under the curve (AUC) for various log-fold changes and different numbers of manipulated genes, see Fig. 2 and the Supplementary Materials. We observed that tissueResolver, BayesPrism, and CIBERSORTx excel in identifying the genes that exhibited differential expression in CD8+ T cells, as evident from the overall steep ROC curves. However, in distinction to any other method, tissueResolver demonstrates the capability to attribute differential expression solely to the CD8+ T-cell compartment, with minimal spill-over to similar cell types like CD4+ T cells or B cells, as only the ROC curve for the CD8+ T-cell compartment displayed a steep incline. The unwanted spill-over was considerably more pronounced in BayesPrism and CIBERSORTx. Especially, BayesPrism identified differential expression of the modified genes across all lymphocyte cell types.

As we increased the artificial fold change, leading to more significant expression changes, it became easier for tissueResolver and BayesPrism to accurately classify the signals, even with a moderate number of selected genes. Interestingly, the increase in fold change also led to a more distinct “misclassification” of BayesPrism for the incorrect cell types CD4 T and B. These conclusions also hold true for different numbers of modified genes and for another cell type being modified, see Supplementary Figs S16–S18.

While tissueResolver does not use cell type labels during fitting, all other algorithms need this information as input prior to the deconvolution. In particular, BayesPrism employs a set of pre-defined cell states, which can be considered very fine-grained cell types. ISLET, CIBERSORTx, and bMIND even require pre-estimated cell type fractions derived from prior deconvolution. In contrast, tissueResolver treats each single-cell profile as an independent reference profile. It is only after the method selects the most suitable cells to explain a tissue that these cells are clustered, and marker genes are utilized to assign them to cell types or cell states. This makes it conceptually different to ISLET CIBERSORTx and bMIND as it estimates cell-type specific expression and proportions simultaneously, see also Supplementary section “Cell type abundance” for proving tissueResolver’s capability of estimating cell type abundance. In practice, these post-modeling analysis options can prove advantageous, as we will demonstrate in the case study on the lymphoma micro-environment in the next section.

## 5 The micro-environment of diffuse large B-cell lymphomas

**Figure 3.**
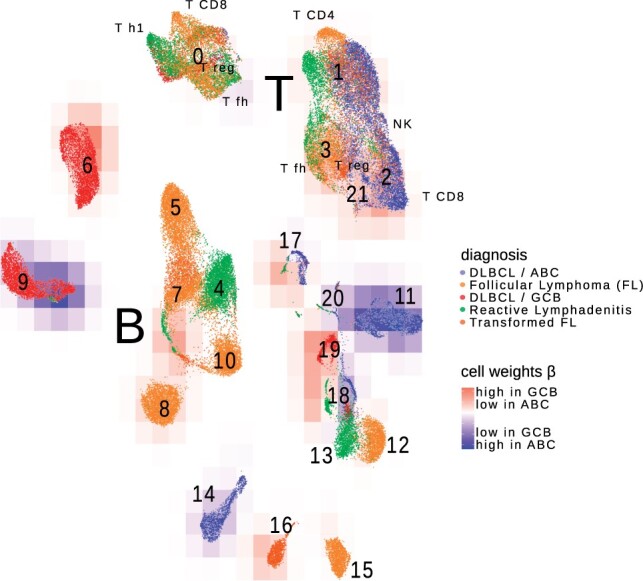
UMAP representation of the single-cell library, colors of the cells indicate the tissue of origin. The cells were incorporated into the virtual tissues of both ABC and GCB lymphomas. A red background indicates that the cells received higher weights in virtual GCB tissues, while blue indicates a preference of the cells in virtual ABC tissues, cf. supplemental Figure 9. Different background colors within a cluster underline the capability of tissueResolver in detecting within cell type heterogeneity.

As a proof of concept, we showcase how tissueResolver explores the gene expression characteristics of tumor tissues. Our case study centers on unveiling differences in the micro-environment of two subtypes of DLBCL, which ressemble different stages of B-cell differentiation: the ABC (activated B-cell like) and GCB (germinal center B-cell like) subtypes (see Alizadeh *et al.* 2000). However, we note that these transcriptionally defined subtypes do not encompass the full heterogeneity of DLBCL and its microenvironment (see e.g. Monti *et al.* 2005, Chapuy *et al.* 2018), and other classifications could be similarly analyzed by our method. We analyzed 481 RNA-seq bulk samples from Schmitz *et al.* (2018). (The data of Schmitz *et al.* (2018) are publicly available in GDC.) Among these samples, 138 belonged to the GCB subtype, and 243 belonged to the ABC subtype. The single-cell library incorporated the combined data from Roider *et al.* (2020) and Steen *et al.* (2021), without further data harmonization steps, allowing tissueResolver to determine the best-fitting profiles. (The scRNA-seq count data of Roider *et al.* (2020) are publicly available under heiDATA ID VRJUNV and EGA accession number EGAS00001004335.) This dataset comprises a total of 63 700 labeled single cells from 20 patients, covering ABC and GCB DLBCL, as well as follicular lymphomas (FL) and benign reactive lymph nodes (rLN), see Supplementary Table S1 and Fig. 3 for further details.

First, we selected the 1000 most variable genes across both bulk and single-cell data (see Supplementary section “Gene filtering”). Subsequently, we ran tissueResolver with 50 bootstrap samples, each consisting of 10% of the cells in the library. On average, virtual tissues comprised 25 cells (standard deviation *σ *= 5) of the 6370 cells available in each bootstrap run. We can interpret these cells picked as representatives of distinct cellular states that tissueResolver recognizes within each bulk sample. In summary, the quality control, as assessed by the scores in “Quality scores and gene selection,” indicated that virtual tissues provided a robust explanation for all bulk samples across the majority of genes, see Supplementary section “Quality control of virtual tissues” for details.

We re-clustered the combined single cells, see Supplementary section “Clustering of single cells.” Figure 3 visualizes the resulting clusters in a UMAP embedding (McInnes *et al.* 2018), while Supplementary Table S2 provides additional information on the composition of the clusters.

Single cells were incorporated into both virtual ABC and GCB tissues, each assigned with distinct weights, represented in Fig. 3 using background coloring. We noticed large cell clusters representing tumor cells that were more prevalently integrated into one tumor subtype compared to the other. Encouragingly, virtual ABC tissues primarily included lymphoma cells from ABC single-cell data, while GCB included cells from GCB and, to a lesser extent, from follicular lymphoma single-cell data. The latter suggests that while follicular lymphomas have expression characteristics different from all DLBCL due to their distinct cellular composition, the malignant cell compartment alone shares similarities with GCB-DLBCL. We also identified small areas within individual clusters showing differential weights between ABC and GCB, indicating nuanced regulatory shifts within sub-populations of a cell type. For instance, in cluster 18 (labeled as non-malignant B cells), some cells increased in weight, while others in the same cluster decreased. Interestingly, this cluster combines cells from two patients—one with DLBCL and one with tonsillitis (T2), as indicated in Supplementary Table S1 and Fig. 3 where we included this patient in the reactive lymphadenitis group. The fact that these cells cluster together supports the annotation as benign B cells. However, the observation that only a subset of these cells was suitable for explaining DLBCL bulks underlines that intra cell type heterogeneity drives the well-known biological heterogeneity of lymphomas (see e.g. Steen *et al.* 2021). Cluster 17 serves as further evidence that even when cell profiles are globally similar and cluster together, tissueResolver can identify differential regulatory effects across bulk tissues within them. The relevance of such fibroblast like clusters was also evidenced in Steen *et al.* (2021) where fibroblasts, subclustered into various states, highly correlated with overall survival.

We proceeded by computing cell type-specific gene expression profiles for all cell types/cell clusters. The ABC/GCB signature was crafted to reflect distinct cells of origin for malignant lymphoma cells (Alizadeh *et al.* 2000, Rosenwald *et al.* 2002, Wright *et al.* 2003). Consequently, we would anticipate that their expression differences are driven by the tumor cell compartment alone. Interestingly, these genes are not exclusively expressed in malignant B cells (Roider *et al.* 2020, Steen *et al.* 2021). Therefore, we employed tissueResolver to untangle the contribution of cell clusters to the overall expression of these signature genes.

**Figure 4.**
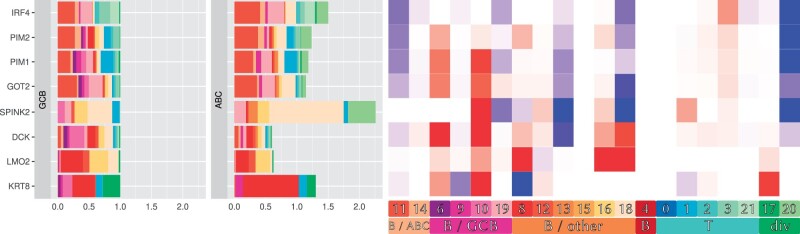
Cell type-specific expression of selected ABC/GCB signature genes (see Supplementary Fig. S2 for the full version) from virtual tissues generated by tissueResolver: The bar plots break down the total expression of a gene into contributions from various cell clusters, indicated by colors and averaged over the ABC and GCB groups, respectively. For better visualization, we normalized the GCB contributions to 1, while the contributions in ABC lymphomas can add up to a higher or lower total amount, depending on the total expression of this gene in this group. The total length of the bar corresponds to the overall fold change of the genes between these groups. The heatmap on the right highlights which cell populations (columns) are responsible for the differential expression of a signature gene (row). Blue indicates increased cell type-specific expression in ABC, whereas red means reduced expression in ABC than in GCB.

Figure 4 presents the outcomes of this analysis for selected genes, for the full signature see Supplementary Fig. S2. The bar plots on the left validate that the signature genes are primarily but not exclusively expressed in tumor cells, with some expression observed in various non-malignant cells. The heatmap on the right attributes the observed expression differences between the two DLBCL subtypes primarily to the malignant B-cell clusters, consistent with expectations.

Several notable exceptions were observed. Firstly, the key regulators IRF4, PIM1, and PIM2 are upregulated in ABC, not only in the lymphoma cells but also in non-malignant plasmablasts (cluster 20), aligning with their recognized physiological role in B-cell fate determination (Gómez-Abad *et al.* 2011, Ochiai *et al.* 2013). Analogously, several other signature genes, e.g. SPINK2, KRT8, DCK, LMO2, and GOT2 showed expression changes attributed to non-malignant cell compartments, such as cluster 17, 20 and the healthy B-cell cluster 18.

We proceed to examine the expression of stromal-1 and 2 signature genes (see Lenz *et al.* 2008). Elevated values of this signature are associated with increased extracellular matrix deposition and infiltration of the tumor by histiocytic immune cells. Thus, the signature is attributed solely to the lymphoma micro-environment. Unlike Lenz *et al.*, we analyze these genes in the context of an ABC/GCB comparison. Many of these genes are in fact differentially expressed between these two subtypes of DLBCL. The majority of signature genes showed upregulation in GCB, and tissueResolver predominantly associated this regulation with cell cluster 17, as shown in Supplementary Fig. S3. Cluster 17 is characterized by high expression of genes in the GCB subtype that are typically found in fibroblast-type cell populations. Our results are in concordance with Steen *et al.* (2021) who also resolved the stromal signature and discovered that the most distinct regulatory effects stem from two endothelial and fibroblast “ecotypes.” In the single-cell data, cluster 17 contained a notably small number of cells compared to the quantities of malignant and non-malignant B cells (Supplementary Table S1). However, despite their limited count, these cells consistently appeared in the virtual tissues, indicating their significance in explaining bulk expressions. This significance is further evidenced in the relative cell weights depicted in Supplementary Fig. S9, where we computed the ratio of mean weights in ABC vs. GCB samples for every cell cluster separately. While we did note slightly reduced weights in the ABC subgroup within cluster 17, these weights alone failed to adequately explain the significant expression variations observed in the stromal genes (Supplementary Fig. S3). Interestingly, this cluster exhibited a substructure: a subset of its cells was predominantly associated with the virtual tissues of ABC tissues, while another subset within the same cluster was predominantly found in GCB tissues. This effect becomes even more apparent when we visualize the differential gene regulation within cluster 17 using a volcano plot (Supplementary Fig. S8a) comparing ABC and GCB gene expression only in cells of cluster 17. Gene labels in Supplementary Fig. S8a highlight stromal signature genes, suggesting that if cluster 17 cells were utilized to define a stromal signature, the resulting gene list would closely resemble the genes identified by Lenz *et al.* (2008). Further details are available in the Supplementary Material.

## 6 Implementation

TissueResolver is implemented as an R package. To compute virtual tissues, it utilizes the L-BFGS-B algorithm (Byrd *et al.* 1995), interfaced through the standard R package optim (R Core Team 2023). This algorithm is capable of handling the box constraints imposed in Equation (1). The tissueResolver package is available at https://github.com/spang-lab/tissueResolver and examples, simulation code, vignettes and code reproducing the results can be found at https://github.com/spang-lab/tissueResolver-docs.

## 7 Discussion

We introduced tissueResolver, a novel approach for estimating cell type-specific expression from bulk expression profiles. The method is entirely regression-based, eliminating the need for a priori cell type definitions, thereby giving full flexibility to the choice of single-cell profiles during the fitting process. Previous current approaches (e.g. Newman *et al.* 2019, Chu *et al.* 2022, Fan *et al.* 2022), accumulate single-cell data to compute cell type-specific profiles, i.e. they learn an expression profile representative for cells of a certain type and then decompose the bulk to obtain abundances of the cell types that were given as an input. The granularity of given cell types determines the output of the algorithms: Only when provided with very fine profiles (corresponding to cell states rather than types) it should become possible to obtain cell type-specific expression, i.e. to observe how the intrinsic “behaviour” of certain cell types changes between conditions. In this sense, computing cell type-specific expression is not much different than estimating cell type abundances (classical deconvolution). However, due to the vast number of possible cell states, the optimization problem is underdetermined and most algorithms break down in this regime of many cell states. Our approach is ultimately fine grained as it gives each single cell, contained in the provided single-cell library, a designated weight. Thus, instead of simply weighting possibly compromised cell type profiles, tissueResolver is capable of tracing differential cell type-specific expression down to the single-cell level, by including only those cells in the virtual tissue whose differentiation state fits the bulk profile.

Not defining cell types upfront is akin to the approach presented in Frishberg *et al.* (2019), with tissueResolver extending these ideas into a comprehensive, parameter-free regression model. BayesPrism (Chu *et al.* 2022) solves the issue of a vast quantity of cell states via a hierarchical approach, by first estimating cell type composition on a coarse level and then refining the estimate using finer cell states. Along with other benchmarked state of the art methods, we see its downside in its reduced performance in our benchmark analysis and in its need to specify cell types up-front.

In combination, our benchmark simulations and our case study underline that in contrast to competing methods tissueResolver is capable of detecting possibly very subtle expression changes as well as attributing them to only the cell compartment sending these regulatory signals. In contrast to many existing approaches, tissueResolver does not rely on population labels of the bulks and so can be applied to identify yet unknown disease phenotypes in an unsupervised way based on the inferred cell-type specific gene expression.

Reconstructing virtual tissues is inherently constrained by the quality of the input data. Specifically, single-cell libraries must encompass cells similar to those in bulk tissues, and technical disparities between single-cell and bulk RNA-seq data should not hinder the reconstruction of the majority of genes. We defined quality metrics that allow to judge fits and interpretability of individual genes.

In summary, tissueResolver emerges as a novel player capable of unveiling concealed information within bulk gene expression data, stemming from various sources, such as solid tumors and blood samples, which enriches our understanding of intra cell type heterogeneity going beyond mere cell abundance. It exhibits flexibility in its application and has shown outstanding performance in benchmark evaluations. In future studies, our method may reveal new prognostically relevant subgroups based on inferred cell type-specific expression of possibly faint cell populations. In this line of thought, tissueResolver can prove beneficial in the discovery of highly targeted drugs.

## Supplementary Material

btae709_Supplementary_Data

## Data Availability

The data underlying this article are available under GEO accession numbers GSE182436 and GSE182434 (single cell data from Steen *et al*. (2021)), under doi:10.11588/data/VRJUNV (single cell data from Roider *et al*. (2020)), and on GDC (DLBCL bulks by Schmitz *et al.* (2018)). Derived, processed data is available on https://zenodo.org/records/10568550.
