# Supplementary material for: Virtual tissue expression analysis
Source: Bioinformatics. 2024 Nov 26;40(12):btae709. doi: 10.1093/bioinformatics/btae709 (PMC11631471; doi:10.1093/bioinformatics/btae709)
Supplement: btae709_Supplementary_Data [file btae709_supplementary_data.pdf]

Bioinformatics, 2024, pp. 1–11

doi: DOI HERE
Advance Access Publication Date: Day Month Year
Paper

PAPER

Supplement to Virtual Tissue Expression Analysis

Jakob Simeth,[1,2]* Paul Hüttl,[2] Marian Schön,[2] Zahra Nozari,[2] Michael Huttner,[2] Tobias Schmidt,[2] Michael Altenbuchinger[3] and Rainer Spang[2]

[1]LIT – Leibniz Institute for Immunotherapy, c/o Universitätsklinikum Regensburg, Franz-Josef-Strauß-Allee 11, 93053 Regensburg, Germany,
[2]Statistical Bioinformatics, Faculty of Informatics and Data Science, University of Regensburg, Am Biopark 9, 93053, Regensburg Germany and
[3]Department of Medical Bioinformatics, University Medical Center Göttingen, Goldschmidtstr. 1, 37077 Göttingen, Germany
*Corresponding author. jakob.simeth@klinik.uni-regensburg.de

Abstract

Supplemental material for "Virtual Tissue Expression Analysis" is provided. In particular we provide details for the DLBCL analysis presented in the main text and the simulations to validate and benchmark tissueResolver. We also present an additional COVID-19 case study.

Gene filtering

Before running tissueResolver, we reduced the number of genes to a common set of 1000 genes of interest to reduce runtime and restrict the optimization to a set of relevant genes. Since we were interested in the interpretation of gene signatures, all available genes from the ABC/GCB signature (1; 2) and the stromal signatures (3) were explicitly included in the final list of genes, irrespective of their expression or variances. The list was then filled up with top variable genes that were determined in the following way: First, ribosomal genes and all genes that were only available in either the bulk or the single cell datasets were removed. Then, the remaining genes were ranked separately by their bulk and single cell variances and the two lists were merged into a single list of joint, highly variable genes, alternating between genes from the single cell and bulk list.

Finally, we used edgeR (4; 5) to compute TMM normalization factors for the bulk on this subset of 1000 genes.

Clustering of single cells

Although single cell datasets provided cell type annotations based on a single cell analysis incorporating prior knowledge, we re-clustered the combined single cell datasets to analyse our computed virtual tissues and compute cell type-specific quantities. For the clustering, we followed the classical Seurat (6) pipeline and used Louvain clustering (7) on the top 15 PCA components with a resolution of 0.5, yielding 22 clusters, see tab. 1 and the UMAP embedding, fig. 3, for the relation to the original cell type annotations.

Differentially expressed genes in virtual tissues

In the main text, we used tissueResolver to give meaning to existing signatures. In this section, we demonstrate that virtual tissues are also useful to explain *why* genes are differentially expressed, i.e., to understand what cell communities are responsible for an observed fold change.

First, we generated a list of differentially expressed genes by using edgeR (4; 5): We used the existing ABC and GCB labels from the Schmitz dataset (10), (re-)computed normalization factors and estimated the dispersion. Since we considered only two groups, we used the classical exact test to compute p-values and false discovery rates. In the following, we restricted ourselves to genes with an absolute fold change greater than $2^{0.8}$ and a false discovery rate below 5 %. Next, we used our virtual tissue to compute cell type-specific gene expression values for the clusters defined above, cf. tab. 2, and selected genes that satisfy our quality criteria of $g_g \leq 0.5$ and $v_g \leq 0.03$, cf. fig. 5 and the discussion in the following section. Cell type-specific expression changes can be read off from fig. 4. We saw that this reveals analogous regulatory effects as considering the classical ABC/GCB signature genes, see section "The micro-environment of diffuse large B-cell lymphomas" with a major overlap in the set of genes that were selected based on the fold change and quality criteria with those from the signature, namely PIM2, BATF, SPIB, SP140, TCF4 and PTPN1. Again, the differences in ABC to GCB are largely attributed to the tumor itself. However, changes in clusters 18 and 20 are also clearly visible, e.g., for FOXP1, PIM2 and BATF.

Quality control of virtual tissues

When screening for genes, it is crucial to judge the quality of virtual tissues and the trustworthiness of results on a per-gene basis. We did so by interpreting the quality scores introduced in section "Quality scores and gene selection". From that section, we recall the gene-specific quality score g_g and the bulk-specific score b_s, cf. eq. (4). Comparing the mean relative residuals of every gene g_g to their respective mean variances $v_g = \frac{1}{N_s} \sum_s \mathrm{var}_k(r_{g,s})$ provides insight to how complete genes can be explained with the available single cell library, see fig. 5 (a): Genes with small residuals that are consistently explained across bootstrap samples (small variance) are fully represented by the single cell reference. Genes with an increased residual are often also less stable. This is an indication that cell identities are missing and the gene expression values in the virtual tissue often deviate from their bulk values. Genes that could not be fit at all ($g_g \approx 1$) show smaller values of v_g, because no cells were

© The Author 2024. Published by Oxford University Press. All rights reserved. For permissions, please e-mail: journals.permissions@oup.com

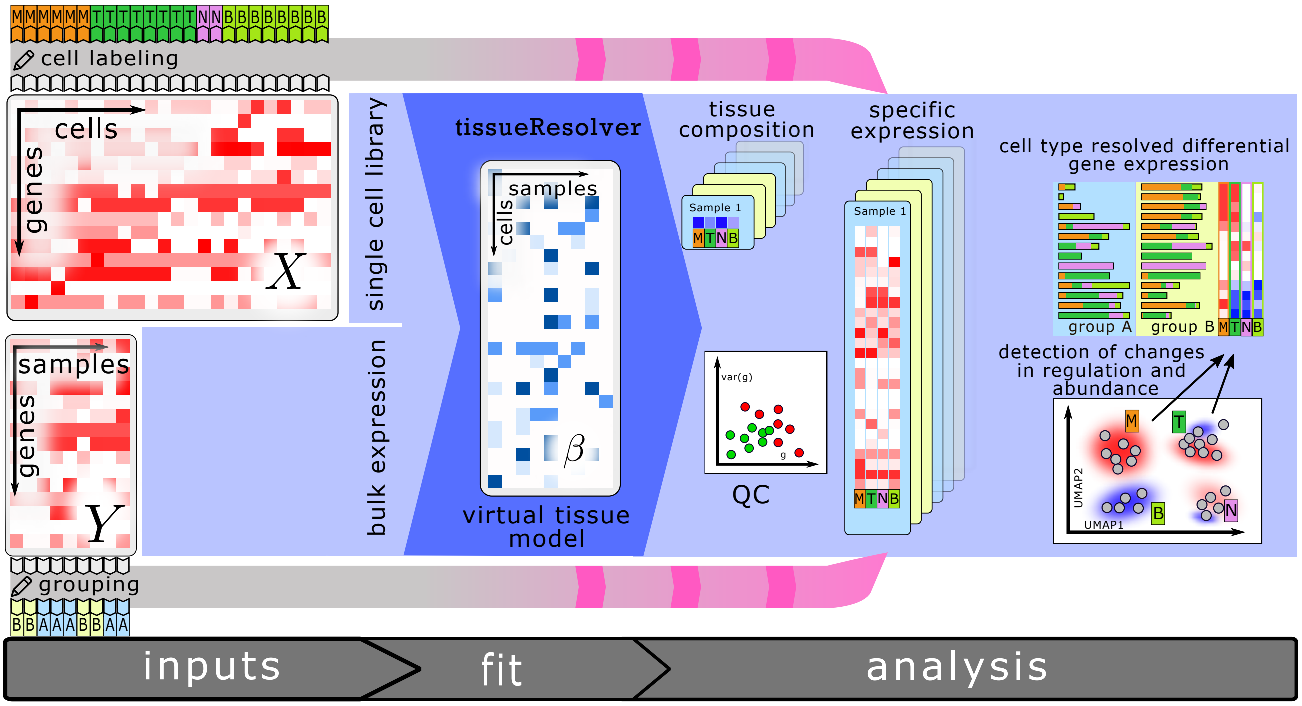

Fig. 1. The tissueResolver workflow: tissueResolver requires two input datasets: Bulk tissue profiles (Y) and single-cell data (X). TissueResolver assigns positive weights (β) to the single-cell profiles attempting to adjust the combination $X\beta$, the fitted tissue, to closely match the bulk expression Y. Finally, the selected cells are grouped by cell type and avaraged to yield celltype specific expression profiles.

id	diagnosis	# cells	B cells: cluster(#)	T cells: cluster(#)	NK: cluster(#)	Plasmablasts: cluster(#)	monocytes: cluster(#)	myeloids: cluster(#)
DCBCL002 (S)	ABC	5534	11(1567),13(80)	1(2594),2(400),3(370)	2(53)	20(44)	17(69)	
DLBCL008 (S)	ABC	3000	11(453)	2(468),1(413),3(71)	2(14)	20(14)		
DLBCL111 (S)	ABC	4400	18(337),13(61),20(49),12(12)	2(1833),1(1382),3(410),21(50)	2(97),1(24)		17(43)	
DLBCL3 (R)	ABC	1963	14(1787)	0(145)				17(21)
DLBCL007 (S)	GCB	1434	19(611),13(206)	2(230),3(163),1(149)				
DLBCL1 (R)	GCB	3120	9(3106)	-				
DLBCL2 (R)	GCB	4277	6(3760)	0(472)				20(15)
tFL1(R)	tFL / GCB	1418	10(294),4(154),5(14),8(12)	0(887)				20(24),17(18)
tFL2(R)	tFL / GCB	2021	7(1520),5(44),4(14)	0(436)				
FL1 (S)	FL	3328	12(1834),13(26)	3(912),2(269),1(245)				
FL2 (S)	FL	3813	15(1687),13(123)	1(841),3(837),2(71)				
FL3 (S)	FL	3078	16(1056),13(19)	1(1042),3(691),2(164)	2(21),1(13)			
FL1(R)	FL	3525	10(2483),4(67),5(26)	0(927)				
FL2(R)	FL	4939	5(3661),7(194),4(65),10(10)	0(1001)				
FL3(R)	FL	4209	8(3133),4(43),10(29),7(15)	0(962)				
FL4 (R)	FL	2986	7(1503),4(799),10(32),8(23)	0(614)				
rLN1(R)	rLN	2454	4(593),0(20)	0(1758)				20(33),17(29)
rLN2(R)	rLN	1722	4(1020),7(180),10(37),5(13)	0(447)				17(10)
rLN3 (R)	rLN	2650	4(1445),7(49),10(19),20(10)	0(1084)				17(20),20(13)
T2 (S)	tonsilitis	3829	13(1355),18(280)	1(1236),3(746),2(145)				

Table 1. Sample identifier of scRNA-seq data of Steen et al. (8) (S) and Roider et al. (9) (R), along with their diagnoses and numbers of cells in each of our clusters. Cluster frequencies below 10 were omitted for brevity. Clusters were assigned to coarse unified labels from the original publications by majority vote.

available that could explain their bulk expression. For these reasons, fig. 4 displays only genes that fulfill $g_g \leq 0.5$ and $v_g \leq 0.03$ and that are differential in the bulks.

Unsurprisingly, genes that showed the smallest relative residuals were also the ones with the highest gene expression in the actual bulk, cf. fig. 5 (b), because they contributed the most to the loss function, eq. (1). These genes that were highly expressed and could be fitted well served as driver genes to tissueResolver and helped in selecting important cells, pinning down their specific weights, whereas cells that did not contribute enough to the loss or co-express genes that would increase the total loss were excluded by assigning small weights. For genes with high relative residuals, in contrast, we typically also observed low bulk expression, with some exceptions, namely genes that were not well covered by the single cell library. Possible reasons for a poor performance in highly expressed genes may also include technological differences between the single cell and bulk RNA-seq workflows.

Lastly, there may also be cases where only a subset of bulks can be fitted sufficiently well. Complete quality control therefore also demands for an assessment of the scores at the bulk level. We considered $r_{g,s}^{(k)}$ averaged now over genes, b_s, and compared the bulk-specific score b_s to the average bootstrap variance $v_s = \frac{1}{N_g} \sum_g \mathrm{var}_k(r_{g,s})$, see fig. 5 (c). In our specific case, the majority of bulks has been fitted with good or acceptable residuals and small variances. If, however, we would have observed high variances, these instable bulks contained significant contributions from cells

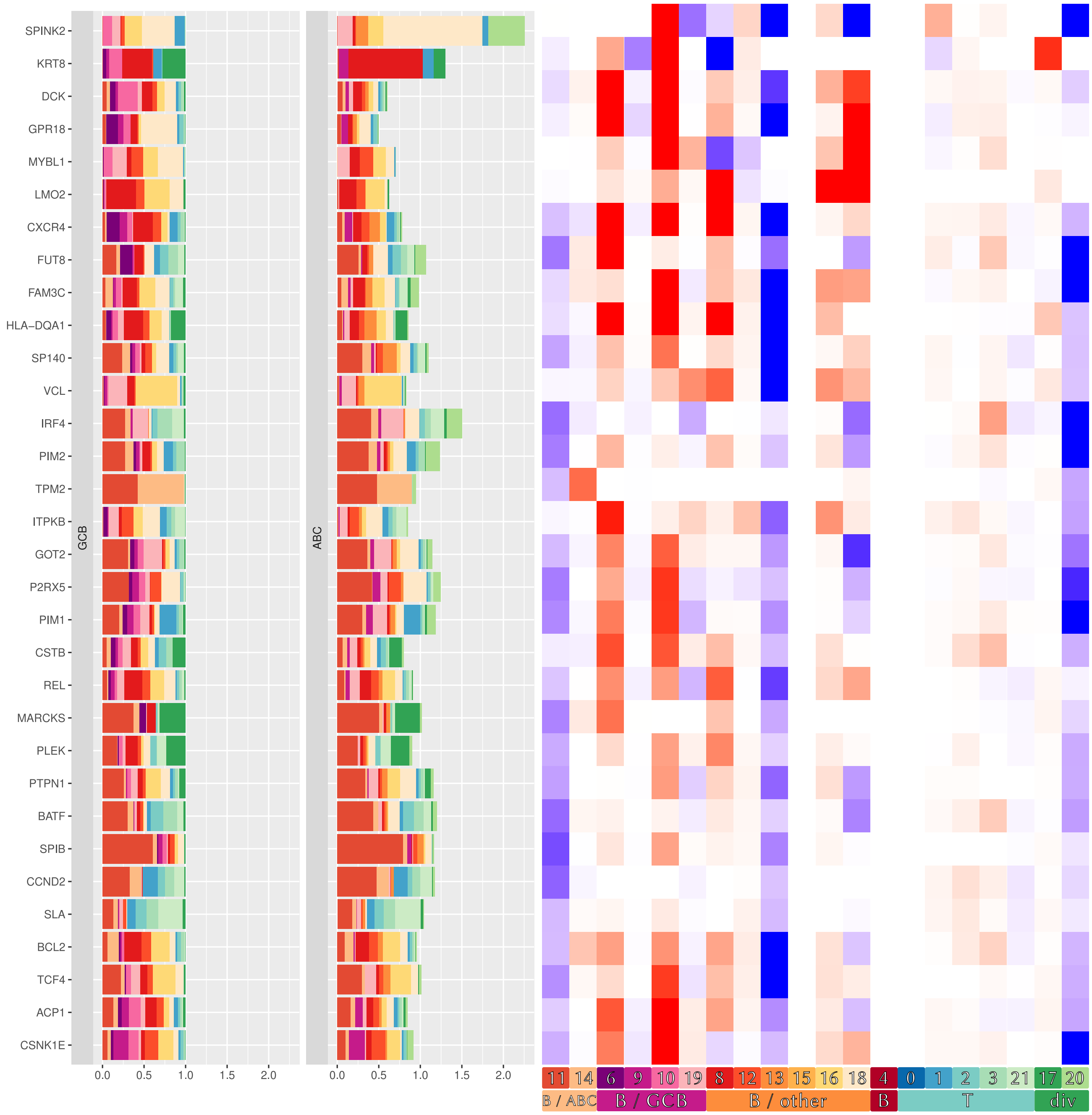

Fig. 2. Cell type-specific expression of ABC/GCB signature genes, see fig. 4 for descriptions.

T+NK	B / ABC	B / GCB	B / all+other	B healthy	myeloid+mono+plasma
0 1 2 3 21	11,14	19,9,6,10,7	12,15,16,5,8,7,18,13	4	17,20

Table 2. Correspondence of cell types to clusters

that are not in our single cell library. By looking at the annotated subtype (10), we deduce that in our case this was not the case and the quality of the fit is almost independent of the tissue's genetic signature and so we did not miss any important phenotypes in our library. Additionally, we see that tissueResolver automatically detected the most fitting cells by selecting cells of (8) to a much higher extent than cells of (9), see fig. 7.

In summary, we saw that virtual tissues computed by tissueResolver are capable of explaining bulk expression as long as the used single cell library contains a reasonable amount of cells essentially covering the heterogeneity of the bulk, e.g., the groups that are compared should also be represented in the single cell data. For the cells of origin of DLBCL constituting the ABC/GCB signature, namely the B-cells, this was the case by observing several patient specific B-cell clusters, see fig. 3 and tab. 1. We stress that

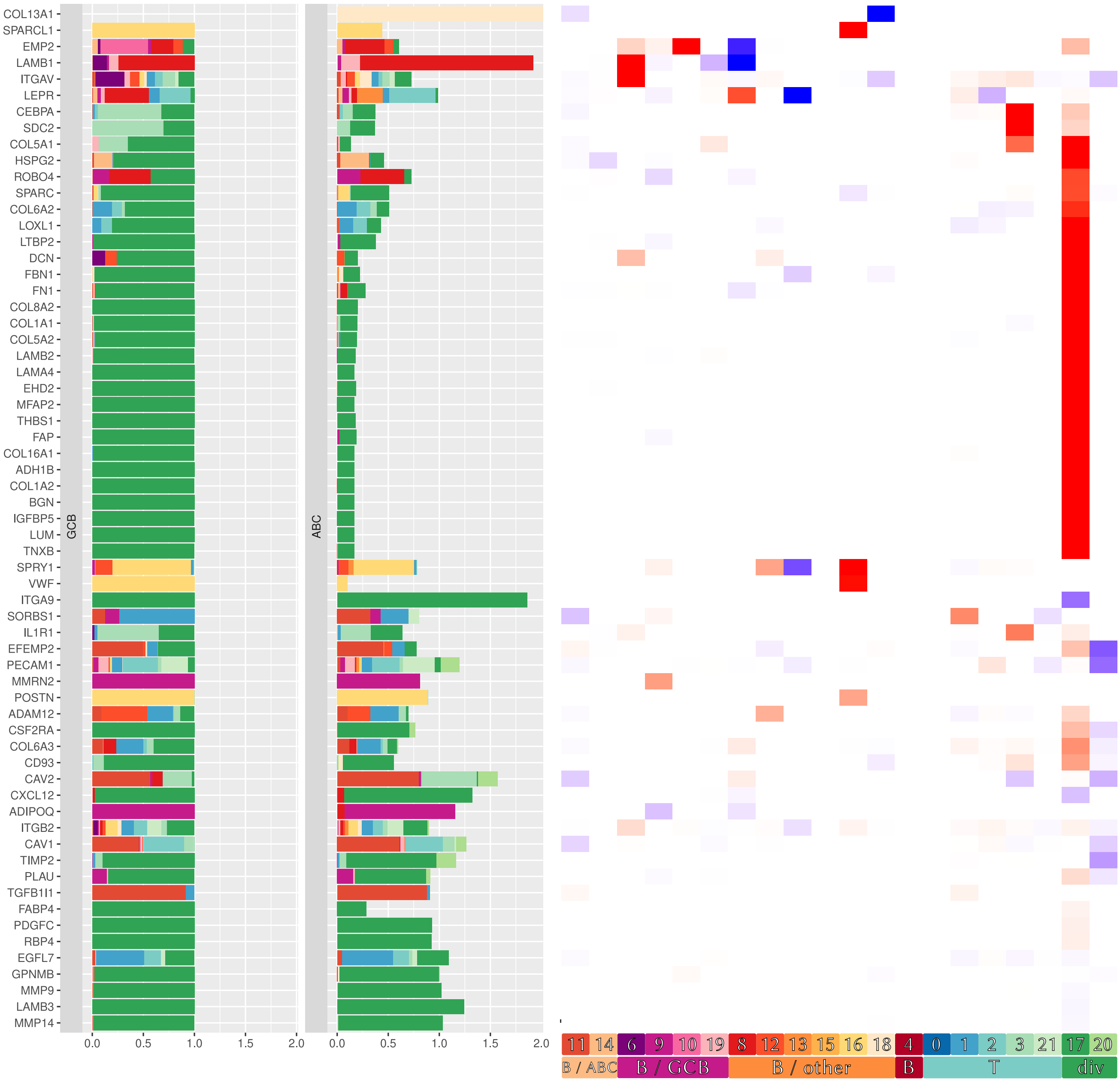

Fig. 3. Cell type-specific expression of stromal signature genes. The figure is organized analogously to fig. 4.

these were different patients than those in the bulk datasets and yet tissueResolver chose a combination of cells from only 20 patients that resembles the bulk expression in a way that it recovered the genetic subtype of the tumors. When screening for genes, however, some caution is required as typically, there exist genes that cannot be fully explained by the available single cells. In the following, we will demonstrate that some information on these genes may still be amenable for interpretation.

Let us reconsider the genes in the stromal signature, cf. fig. 3. We show the quality scores for these genes in fig. 6. First of all, we saw that many of these genes were only moderately expressed in the bulks and showed a high relative residual, i.e., using the available single cell data, the virtual tissues could not fully explain the entire gene expression of the bulks. However, we observed, that many of these genes were attributed to cluster 17 and behaved stable across all bootstrap runs (small v_g). Also, cluster 17 was assigned stable weights across the two groups and the total sum of weights changed only slightly between them, cf. fig. 9. In other words: The cell weights in cluster 17 that were chosen to explain the bulks were pinned down precisely by highly expressed driver genes, cf. fig. 4 but also fig. 6 b) and c) where cluster 17 contributed significantly to highly expressed genes with good quality scores.

One of the key features of tissueResolver is that the reference matrix consists of true, actual cells. Therefore, although the bulk expression was not fully explained (large g_g), the *contribution* of cluster 17 to the total expression can be trusted because in that cluster these genes were co-expressed with genes that allowed to predict the frequencies and weights of the relevant cell population. The integrity of the cells' profiles allowed us to view the resulting tissue as a predictor also for the lowly expressed genes of the stromal

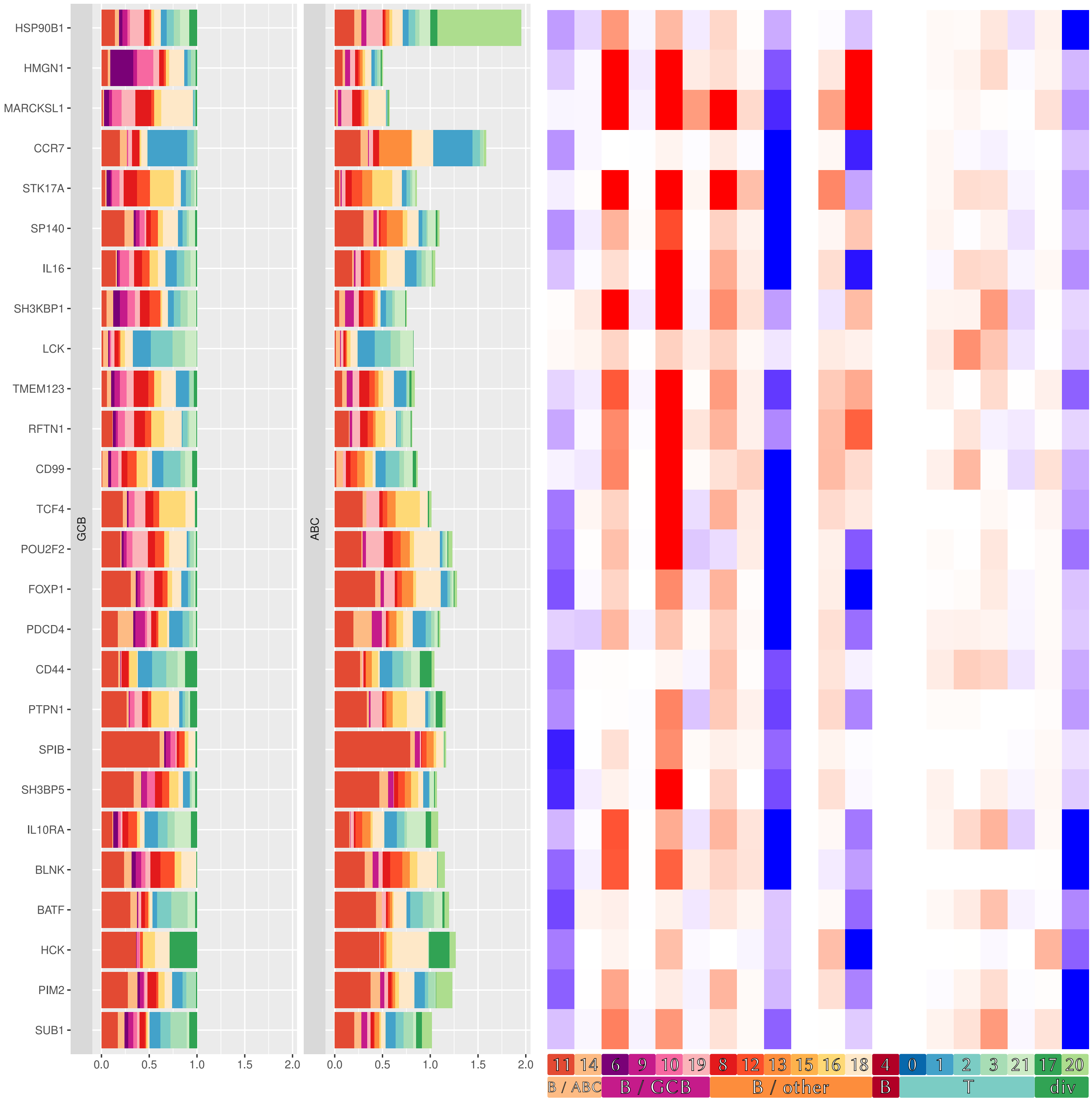

Fig. 4. Cell-type specific expression of genes with absolute log fold change $> 2^{0.8}$, FDR ≤ 0.05, a relative residual $g_g \leq 0.5$, and an average variance of the relative residual $v_g \leq 0.03$. See fig. 4 for descriptions.

signature. This was only possible as our algorithm is not demanding for a priori cell clustering, thus, giving each single cell an unbiased chance to show its relevance in differentiating between genetic signatures and is thus, independent of the cell type consistency constraint of (11).

With this in mind, we were tempted to have a closer look at individual clusters that were consistently integrated into virtual tissues. For instance, when we aim to examine differences in cell type-specific gene expression across two classes of tissues, it is essential to account for variations in the abundance of these cells in each tissue. To achieve this, the cell type-specific expression is straightforwardly normalized by its relative weight, c_s^a,

$$R^a_{\cdot,s} = \frac{1}{\sum_i \beta_{i,s}} \frac{\widetilde{Y}^a_{\cdot,s}}{c^a_s}, \tag{1}$$

where the additional normalization factor accounts for different library sizes across bulks. In fig. 8 (a), we show fold changes and corresponding false discovery rate between the two conditions in a classical volcano plot, but only using the specific regulation found in cluster 17. Comparing the differential cell type-specific regulatory genes with those in the stromal signature (labels in the figure), we found that the signature was well recovered by these cells and, had we screened for a cell type-specific signature in our data, we would

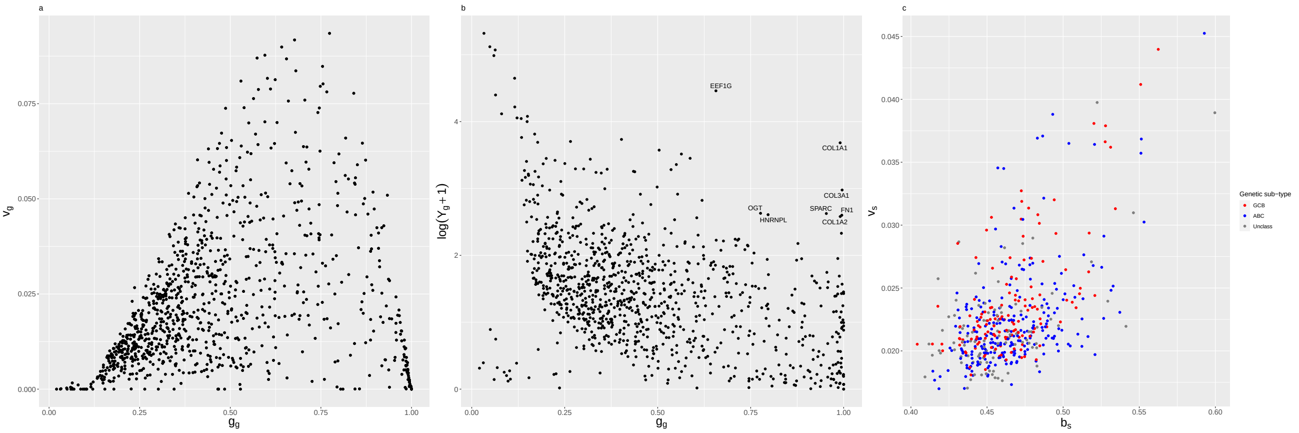

Fig. 5. (a) Mean relative residuals, eq. (4), for every gene g_g and their average bootstrap variance, $v_g = \frac{1}{N_s}\sum_s \text{var}_k(r_{g,s})$. High variance values may indicate missing cell types in the reference.
(b) Mean relative residuals g_g and average expression $\log(\sum_s \frac{Y_{g,s}}{N_s} + 1)$ of genes. Highly expressed genes are typically well covered by the fit and display small relative residuals, whereas many of the lowly expressed genes are below the noise level and can hardly be interpreted. Genes that are highly expressed but show a large deviation indicate expression in cells that are not present in the single cell library.
(c) Mean relative residuals for every bulk b_s and their average bootstrap variance, $v_s = \frac{1}{N_g}\sum_g \text{var}_k(r_{g,s})$. High variance values may indicate bulk tissues comprised of cells that are missing in the reference. We additionally colored each sample according to its genetic subtype. The equal distribution of colors indicates that there were no subtype specific differences in the quality of the fit.

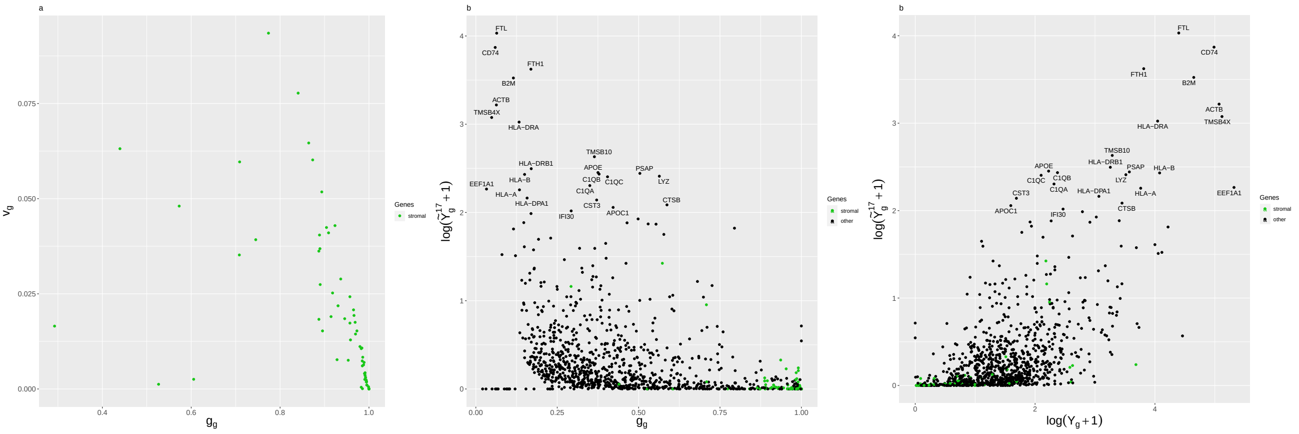

Fig. 6. (a) Quality scores for stromal genes. See fig. 5 for details. Subfigures (b) and (c) show the mean expression of each gene declared by cluster 17. We see that there are genes which are highly expressed both in cluster 17 and the actual bulk, which at the same time possess low relative residuals.

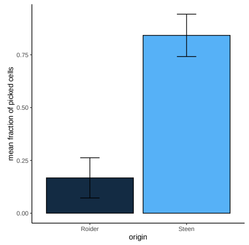

Fig. 7. Average composition of virtual tissues when employing the combined single cell library of (8) and (9) in the fit. Error bars indicate the standard deviation across bulk samples per origin group.

likely have ended up with a similar result as Lenz et al. (3). To underline this predictive strength of our algorithm we additionally considered in fig. 8 (b) the analogous plot for the benign B-cell cluster 18 which revealed some genes of the ABC/GCB signature among its differentially regulated genes. This highlights the use of tissueResolver as a tool to identify cellular mechanisms and signatures that would otherwise remain hidden in the bulks.

Expression differences in COVID-19 patients

Expression changes in the tumor microenvironment may sometimes be quite drastic and localized around the solid tumor, leading to pronounced and characteristic signals. In this section we apply tissueResolver to a completely different, more subtle situation: We compared CD4 T-cells extracted from the blood of COVID-19 patients that were hospitalized in an intensive care unit (ICU) with those of patients showing no or only mild symptoms (control). We re-analyzed the CD4 bulk samples from (12) and compared four bulks in the control with four in the ICU group. To compute virtual tissues, we used single cell data published in (13) that has been subsetted, preprocessed and clustered as described in (12), where some of the authors have estimated cell-cluster abundances of the bulks using DTD (14) with the same single cell data. Using tissueResolver, again, clusters 9 and 2 turned out to be the dominant clusters

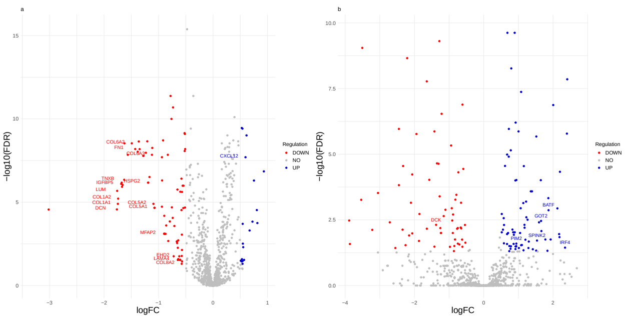

Fig. 8. Volcano plot of cell type specific gene regulation in cluster 17 (a) and 18 (b) respectively. Genes with absolute fold change higher than 0.5 and false discovery rate less than 0.05 appear in color. Among those we additionally labeled genes which belong to the stromal signatures in a) and genes which belong to the ABC/GCB signature in b).

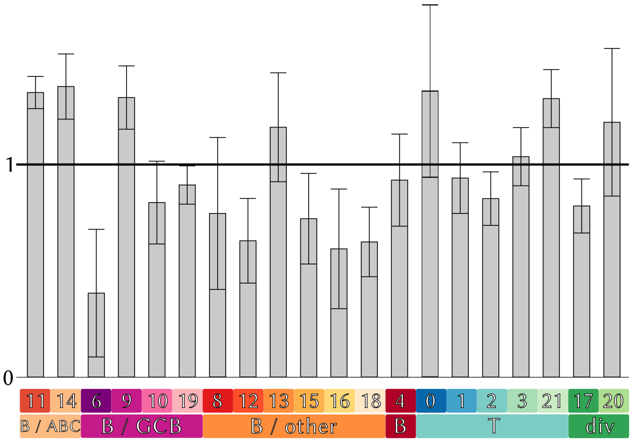

Fig. 9. Estimated cellular composition of ABC vs. GCB tissues. Shown are ratios of average cluster weights between the two DLBC subtypes.

responsible for the bulk of the expression, see fig. 10, whereas most other clusters were not found at all or contributed very little to the total expression. In (12), cluster 2 was found to be expressing hypoxia related genes, and to be more present in the ICU group. tissueResolver confirmed this finding, see fig. 0.2, mainly due to a slightly higher overall abundance of the respective cell cluster, see fig. 10. The frequency of cluster 9, however, was constant across conditions and the tissueResolver result (fig. 10) confirmed the original publication's DTD finding. Due to this non-differentiality in terms of abundance, this cluster was not considered further in (12). The use of tissueResolver revealed, however, that on an expression level cluster 9 changed quite drastically between the two conditions (fig. 11) and also in this cluster many genes associated to cell metabolism were differentially expressed between the groups, e.g. SLC7A5, SLC2A3, ABCG1, LDLR, TXNIP, DGKA, PDE7A. Some of the genes have been shown to be directly related to COVID-19, e.g., TXNIP is involved in oxidative stress as well as inflammation regulation and servses as a potential therapeutic target (15). The role of cluster 9 was completely missed in the original publication, because classical deconvolution algorithms are unable to detect changes in gene regulation.

This example also illustrates an important feature of tissueResolver: Although some clusters received very low overall weights, they still were picked consistently in the fits and reduced the residual value. The reason for this lies in individual genes whose residual value was improved drastically by including them, see, e.g. clusters 11

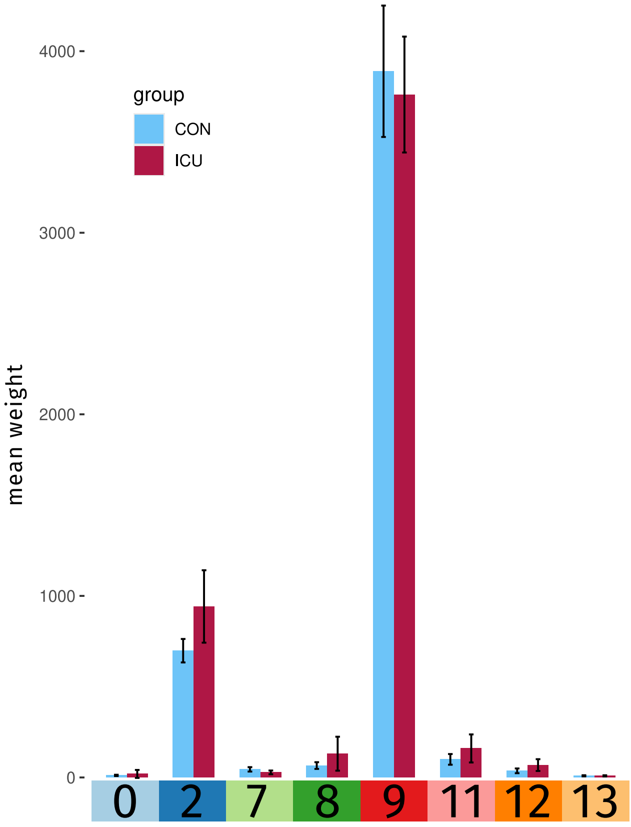

Fig. 10. Estimated abundances of CD4 T-cell clusters in COVID-19 patients for the control and ICU groups.

and 13 in fig. 0.2 for genes MT1E and IPCEF1, respectively, or also fig. 11 for the glycolysis related gene ENO1. We conclude that tissueResolver is very sensitive to even subtle expression changes and can identify even rare cell communities in the bulk.

Scaling behaviour and selection of bootstrap bin sizes

In this section we investigate how tissueResolver deals with different problem sizes, i.e., we varied the percentage of single cells used in each fit, and saw how this affects computing time. This is an important information for selection of bootstrap bin sizes. To this end, we repeated the fit from the previous section, but varied the bootstrap size. The COVID-19 dataset (13) consists of 1.5 million cells and even after filtering, the remaining 127,036 CD4 positive cells provided a solid basis for this test.

As expected, we saw that the more cells are available for the fit to choose from, the more fine-tuning was possible to achieve a small residual and more and more cells were added to the set of selected cells (cells with non-zero weight). However, as also indicated in the main text, this came at the cost of overfitting to potentially spurious cells. From the elbow shape in fig. 13 c) we saw that for a bootstrap bin size of more than 10% the gain in relative residual saturated, indicating that this bin size is a sweet spot balancing the trade-off between overfitting and low residuals. At this point, also the bootstrap variance decreased, indicating overfitting (fig. 13d)). In general, the number of cells where the residual is good, but no overfitting takes place, will depend on the heterogeneity of the single-cell library.

To no surprise, providing many cells also increased the runtime of tissueResolver. Fig. 13 b) asymptotically declared a power

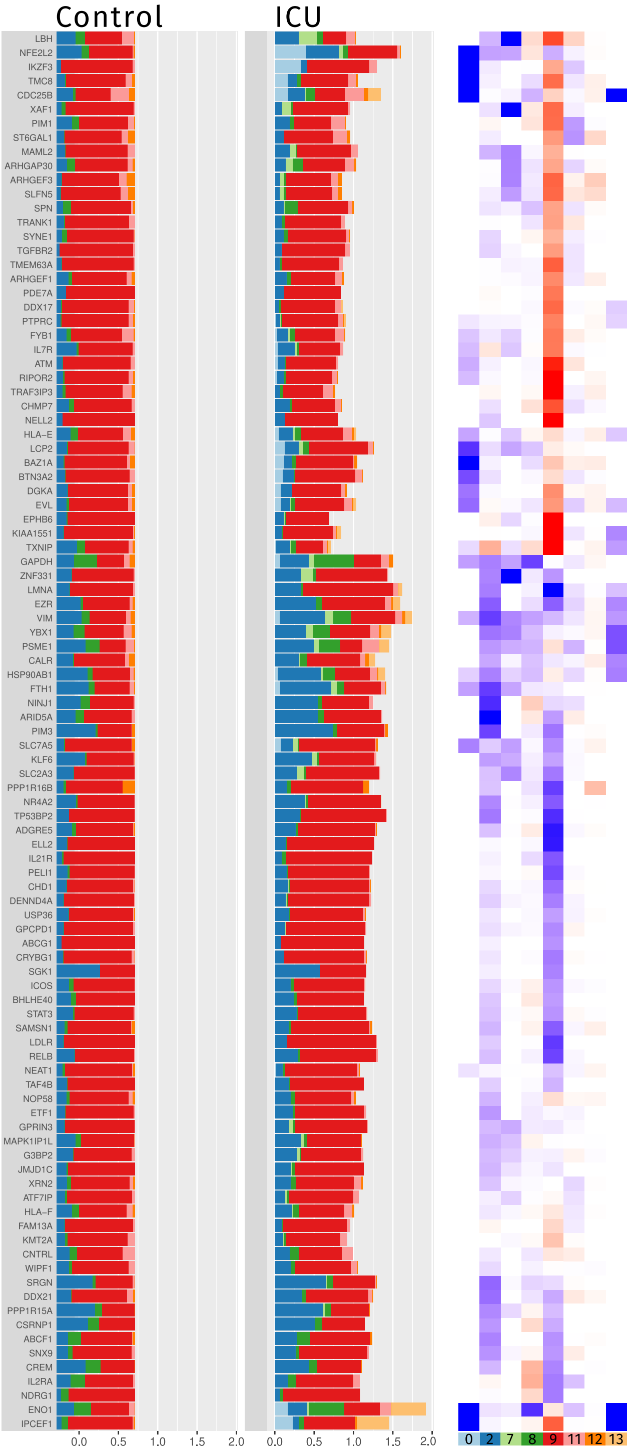

Fig. 11. Estimated gene expression of CD4 T-cell clusters in COVID-19 patients. The figure is organized similarly to fig. 4. We show genes with log_2-foldchange greater than 0.5 with $p < 0.05$ and a relative residual better than 0.5.

(exponent $\approx$ 1.5) relation between the duration of fitting a bulk with tissueResolver and the size of the single cell library. When we let tissueResolver pick cells from the full set of 127,036 cells, each bulk took approximately one core-hour of computing time – a considerable amount that again motivates subsetting the cells

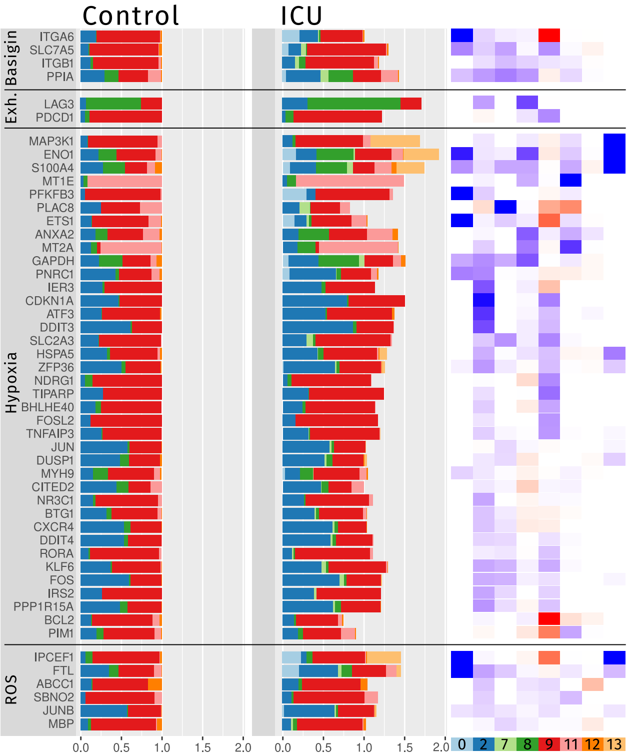

Fig. 12. Estimated expression of CD4 T-cell clusters in COVID-19 patients. The figure is organized similarly to fig. 4. Gene sets taken from the original publication (12).

into smaller bootstrap bins. We note that the computation time depicted here is for a single threaded run of tissueResolver and the implementation allows to fit to several bulks in parallel, reducing overall time-to-solution.

In conclusion, a large-enough number of cells must be present to fit the bulk consistently, but the choice of the bootstrap bin size is relatively stable across a wide range. Computing time can be reduced by picking a smaller bin size and in turn repeating the fit several times (by increasing the number of bootstrap runs).

Simulation details

In the following we provide our test setup as pseudo code, algorithm 1, providing additional details and a step by step pipeline supplementing the explanations in section“Simulations”. Note that for BayesPrism, we included two possible scenarios, one where we used exclusively the annotated cell types from the single cell dataset (“bp-nosub”, step 4 (b) and one where we subclustered every annotated cell type (“bp-sub”, step 4 (c), also see section “Clustering of single cells” for details on the clustering) and provided these as “cell states”. For ISLET (16), bMIND (17) and the HiRes module of CIBERSORTx (18) we used the default parameters as indicated in the respective documentations. For CIBERSORTx we made use of the cibersortx/hires docker container using S-mode batch correction for determining a signature matrix from single cell data and used B-mode batch correction when employing this signature matrix in the deconvolution of bulk samples. We stress that these algorithms require previously estimated cell type proportions as input. Thus, we followed all algorithms’ recommendations and use CIBERSORT (18) for ISLET and the HiRes module, and Bisque (16) for bMIND,

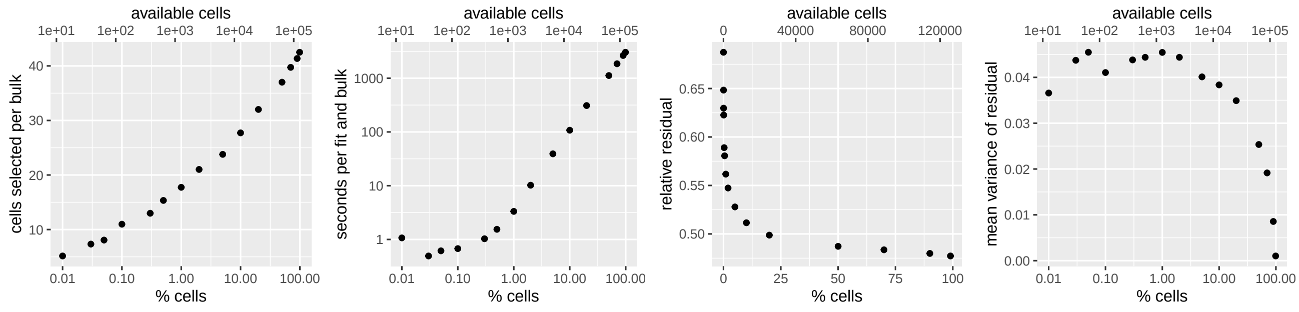

Fig. 13. Selected number of cells, time to fit one bulk profile, relative residual, and mean variance of the residual, respectively, as a function of the bootstrap bin size. The upper horizontal axis indicates the number of cells that are included in each bin.

respectively. [1]
Additionally, we note that both bMIND and ISLET are algorithms designed to include population level information into the deconvolution, whereas BayesPrism and tissueResolver work on single bulk profiles and do not require repeated or temporal measurements.
The simulation was repeated five times to determine averages and standard errors of fig. 2 (step 5 in algorithm 1) resulting from random sampling when constructing artificial bulks. Runnable R code for all benchmarked algorithms as well as the tissueResolver benchmarks is provided along with the vignettes on `https://github.com/spang-lab/tissueResolver-docs`.

The selection of genes that were modified, $\mathcal{G}_{\mathrm{mod}}$, was done in the following way: From all annotated CD8+ T-cells, we determined the set of genes that showed non-zero expression in at least 40 % of all these cells and further required that the genes are also expressed in 40 % of all available cells (across cell types). This avoids the selection of highly specific genes that would result in a trivial deconvolution task, as the genes differentiating between the two bulk groups were then only expressed in the cell type we have modified and acted as marker genes.

Complementing the modification of the CD8 T-cell compartment, we performed the simulation again, but now modifying only cells from the B-cell compartment, which in the artificial mixtures was the most abundant cell type. The dataset (8) comprises 9915 B-cells of which 6479 enter our source for constructing bulk mixtures, which is more than five times the amount of CD8 T-cells, which only make 1157 cells in our bulk source. Thus, as opposed to the possibly faint (absolute) changes in CD8 T-cells, we consequently introduced more pronounced changes in terms of absolute gene expression. We challenged tissueResolver and its competitors to recognize cell type-specific gene expression and attribute it to the B-cell compartment. The results of this benchmark are depicted in fig. 16 and fig. 18. Fixing a log fold-change of 0.8 and 39 modified genes in fig. 16 we noted, that as expected, the detected cell type-specific differential expression is more pronounced regarding the ROC curves. Concerning BayesPrism this introduced also a more severe spillover to non-differentially expressed cell types. TissueResolver again attributed the changes in expression solely to the B-cell compartment with only minor spillover. CIBERSORTx and bMIND attributed differential expression to the correct compartment, but we also observed distinct spillover. Note that CIBERSORTx could not explain differential CD4 T expression as all profiles estimated by the HiRes module were identical, and thus no ROC and AUC curves are depicted for this compartment. Concerning ISELT, we observed a drastic performance increase compared to the prior CD8 T benchmark. It is now on par with tissueResolver in the sense that it possesses both correct attribution and minimal spillover.

Looking at the second row in fig. 16 we saw that these conclusions were also inherited by different fold-changes, with an increased performance of tissueResolver and ISLET for more pronounced log fold-changes. Note that the last fold-change in CIBORSORTx is missing for the B-cells as the profiles were almost constant without significant variation. Interestingly, fig. 18 revealed that CIBERSORTx, bMIND, and ISLET reacted very sensitive to varying numbers of modified genes, whereas tissueResolver provided superior performance independently of the number of genes modified.

Cell type abundance

Traditionally the main task of deconvolution tools is to give an estimate on the abundance of cell types within a tissue in order to detect cell-type specific cross sample heterogeneity in terms of relative proportions. We stress that the main task of tissueResolver is to estimate cell type-specific expression. However, due to the weighting of each single cell in the reference library the algorithm naturally provides an estimate on cell proportions, see also section "Cell frequencies and cell type-specific gene expression from virtual tissues". Thus, in order to give consistent expression estimates, the cell proportions explained by the virtual tissues are expected to be close to the ground truth cell type abundance within each tissue. In order to test whether tissueResolver is capable of explaining cell type-specific proportions and in order to compare its performance against other state of the art algorithms we performed the following benchmark conceptually similar to the simulation layout described in section "Simulations".

We again considered the single cell data set of (8) and randomly divided the eight patients into two groups in order to constitute an independent single cell library and a source for generating artificial bulks. However, as opposed to section "Simulations", we now composed artificial bulks on the patient level rather than intermixing cells from different patients. More precisely, in order to generate a bulk mixture we summed up over cells stemming from a *single* patient in order to capture a patient's biological integrity, as also suggested in (11, Supplementary Note 1). However, in order to introduce artificial variability between these bulk samples we did not simply pick all cells constituting a patient, but for each cell-type

[1] Note that bMIND also gives the option of estimating prior information from the single cell data, however, on our dataset the covariance matrix for each gene was computed to be negative definite by `get_prior` from the `MIND` package, which prohibited us from including prior information into bMIND.

Algorithm 1 Simulations for benchmarking tissueResolver and BayesPrism

Require: $X \in \mathbb{R}_+^{N_g \times N_c}$ ▷ sc dataset
Require: $\mathcal{T} \leftarrow \{j \in \{1, \ldots, N_c\} \,|X_{\cdot,j} = \text{CD8 T-cell}\}$
Require: $\mathcal{G}_{\text{mod}} \subset \{1, \ldots, N_g\}$ ▷ genes to be modified
Require: $\gamma \in [2^{0.5}, 2^2]$ ▷ foldchange

Step 1: Split cells into reference and bulk simulation sets
Require: $\mathcal{P}_1 \cup \cdots \cup \mathcal{P}_8 = \{1, \ldots, N_c\}$ ▷ group by patients
$\mathcal{Q}_{\text{bulk}} = \text{SAMPLE}(\{\mathcal{P}_1 \ldots \mathcal{P}_8\}, 4)$ ▷ 4 patients to build
$\mathcal{Q}_{\text{ref}} \leftarrow \{1, \ldots, N_c\} \setminus \mathcal{Q}_{\text{bulk}}$

Step 2: Modify selected genes in half of CD8 T-cells
$\mathcal{T}_{\text{mod}} \leftarrow \text{SAMPLE}(\mathcal{T}, \frac{|\mathcal{T}|}{2})$ ▷ sample half of CD8 T
for $j \in \mathcal{T}_{\text{mod}}$ **do**
  for $i \in \mathcal{G}_{\text{mod}}$ **do**
    $X_{i,j} \leftarrow \gamma X_{i,j}$ ▷ modify by foldchange
  end for
end for
$X^{\text{ref}} \leftarrow X_{\cdot,\mathcal{Q}_{\text{ref}}}$

Step 3: Build artificial bulks of two conditions
$\mathcal{Q}_{\text{bulk}}^{\text{unmod}} \leftarrow \mathcal{Q}_{\text{bulk}} \setminus \mathcal{T}_{\text{mod}}$ ▷ set w/ unmodified CD8+T
$\mathcal{Q}_{\text{bulk}}^{\text{mod}} \leftarrow \mathcal{Q}_{\text{bulk}} \setminus (\mathcal{T} \setminus \mathcal{T}_{\text{mod}})$ ▷ set w/ modified CD8+T
for s=1,...,50 **do**
  $\mathcal{B} \leftarrow \text{SAMPLE}(\mathcal{Q}_{\text{bulk}}^{\text{unmod}}, 500)$ ▷ randomly select cells
  $\omega \sim [\mathcal{N}(\mu = 1, \sigma = 0.3)]^{500}$ ▷ Gaussian weights
  $Y_{\cdot,s}^{\text{unmod}} \leftarrow \sum_i \omega_i X_{\cdot,\mathcal{B}_i}$
end for
for s=1,...,50 **do**
  $\mathcal{B} \leftarrow \text{SAMPLE}(\mathcal{Q}_{\text{bulk}}^{\text{mod}}, 500)$ ▷ randomly select cells
  $\omega \sim [\mathcal{N}(\mu = 1, \sigma = 0.3)]^{500}$ ▷ Gaussian weights
  $Y_{\cdot,s}^{\text{mod}} \leftarrow \sum_i \omega_i X_{\cdot,\mathcal{B}_i}$
end for
$Y \leftarrow [Y_{\cdot,s}^{\text{mod}}, Y_{\cdot,s}^{\text{unmod}}]$ ▷ combine bulk groups

Step 4a: Fit the artificial bulks with tissueResolver
$\beta \leftarrow \text{TISSUERESOLVER}(Y, X^{\text{ref}})$
for $a \in \mathcal{M}$ **do**
  $\widetilde{Y}_{\cdot,s}^{a} = \sum_{\substack{i \\ l_i = a}} X_{\cdot,i}^{\text{ref}} \beta_{i,s}.$
end for

Step 4b: Fit the artificial bulks with BayesPrism (no subtypes)
$\widetilde{Y} \leftarrow \text{BAYESPRISM}(Y, X^{\text{ref}}, l)$

Step 4c: Fit the artificial bulks with BayesPrism (with subtypes)
for $a \in \mathcal{M}$ **do**
  $l^{\text{fine}} = \text{CLUSTER}\{X_{\cdot,i}^{\text{ref}} | l_i = a\}$
end for
$\widetilde{Y} \leftarrow \text{BAYESPRISM}(Y, X^{\text{ref}}, l, l^{\text{fine}})$

Step 4d: Fit the artificial bulks with CIBERSORTx
$X^{\text{sig}} \leftarrow \text{CIBERSORTx}_{\text{fractions,S-mode}}(Y, X^{\text{ref}})$
$\widetilde{Y} \leftarrow \text{CIBERSORTx}_{\text{HiRes,B-mode}}(Y, X^{\text{sig}})$

Step 4e: Fit the artificial bulks with bMIND
$c \leftarrow \text{BISQUE}(Y, X^{\text{ref}})$
$\widetilde{Y} \leftarrow \text{bMIND}(Y, c)$

Step 4f: Fit the artificial bulks with ISLET
$X^{\text{sig}} \leftarrow \text{CIBERSORTx}_{\text{fractions,S-mode}}(Y, X^{\text{ref}})$
$c \leftarrow \text{CIBERSORTx}_{\text{fractions,B-mode}}(Y, X^{\text{sig}})$
$\widetilde{Y} \leftarrow \text{ISLET}(Y^{\text{mod}}, Y^{\text{unmod}}, c)$

Step 5: Repeat steps 3 to 4 to gather statistics

and each artificial bulk separately, we sampled from the distribution

$$\min(|\mathcal{N}(1, 1)|, 1.5),$$

and then drew the resulting proportion of cells (with replacement). So sampling a factor 1 of above distribution would imply taking exactly the amount of cells of the specific cell type as actually measured for the patient.
Furthermore, for consistency between bulk source and single cell library we restricted to those cell types contained in every patient, namely B, CD8 T, CD4 T and T follicular helper.

As described in (11, Methods) and (19, Supplementary Note 4) the ground truth cell type abundance in bulk sample s of cell type a, $c_s^{a,\text{True}}$, can be computed as the fraction of total reads within a cell type-specific expression profile Y_s^a over the total reads in the bulk expression Y_s, i.e.,

$$c_s^{a,\text{True}} = \frac{\sum_{i=1}^{N_g} Y_{i,s}^a}{\sum_{i=1}^{N_g} Y_{i,s}} \in [0, 1]. \tag{2}$$

We generated 100 artificial bulk mixtures and let several cell proportion estimation algorithms deconvolve these tissues. We repeated this simulation 5 times, with different random sampling in each step, i.e., the initial patient split and thus the single cell library as well as the source for simulating bulks were different in each run.
We benchmarked tissueResolver against BayesPrism (with prior cell state subclustering), Bisque (20), CIBERSORTx (18), DTD (14) and MuSiC (11). For Bisque we used the default settings as interfaced in the `MIND` R package of bMIND (17), for CIBERSORTx we employed the docker cibersort/fractions module with default parameters for determining a signature matrix from the single cell library using S-mode batch correction, for DTD we trained on half of the bulk mixtures and tested on the other half (in all benchmarks we thus report only on the test mixtures) and for MuSiC we also use default parameters. Note that the two step iterative deconvolution in MuSiC2 (21) is equivalent to the initial MuSiC (11) implementation, as we do not include different sample conditions.

We present the quality metrics as computed in (11, Methods) in fig. 14 and fig 15. From fig. 14 we deduced that tissueResolver is only slightly inferior to BayesPrism, but yields major improvements to competing methods in all quality metrics. Considering the actual explained proportions in comparison with ground truth proportions we again observed that BayesPrism and tissueResolver excel in explaining cell type specific abundance, see fig. 15. We concluded, that tissueResolver excels most its competitors, being almost on par with BayesPrism.

References

1. Alizadeh, A. A., Eisen, M. B., Davis, R. E., Ma, C., Lossos, I. S., Rosenwald, A., Boldrick, J. C., Sabet, H., Tran, T., Yu, X., et al. (2000). Distinct types of diffuse large b-cell lymphoma identified by gene expression profiling. *Nature*, 403(6769):503–511.
7. Blondel, V. D., Guillaume, J.-L., Lambiotte, R., and Lefebvre, E. (2008). Fast unfolding of communities in large networks. *Journal of statistical mechanics: theory and experiment*, 2008(10):P10008.
19. Chu, T., Wang, Z., Pe'er, D., and Danko, C. G. (2022). Cell type and gene expression deconvolution with bayesprism enables bayesian integrative analysis across bulk and single-cell rna sequencing in oncology. *Nature Cancer*, 3(4):505–517.
21. Fan, J., Lyu, Y., Zhang, Q., Wang, X., Li, M., and Xiao, R. (2022). Music2: cell-type deconvolution for multi-condition bulk rna-seq data. *Briefings in Bioinformatics*, 23(6):bbac430.

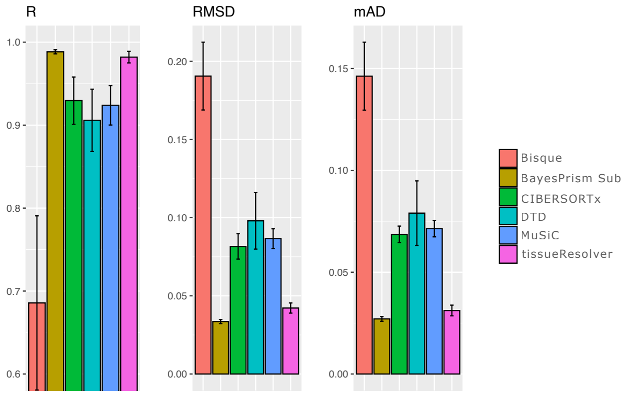

Fig. 14. Summary statistics for cell type abundance benchmarking, as introduced in (11).

16. Feng, H., Meng, G., Lin, T., Parikh, H., Pan, Y., Li, Z., Krischer, J., and Li, Q. (2023). Islet: individual-specific reference panel recovery improves cell-type-specific inference. *Genome biology*, 24(1):174.
14. Görtler, F., Schön, M., Simeth, J., Solbrig, S., Wettig, T., Oefner, P. J., Spang, R., and Altenbuchinger, M. (2020). Loss-function learning for digital tissue deconvolution. *Journal of Computational Biology*, 27(3):342–355. PMID: 31995401.
20. Jew, B., Alvarez, M., Rahmani, E., Miao, Z., Ko, A., Garske, K. M., Sul, J. H., Pietiläinen, K. H., Pajukanta, P., and Halperin, E. (2020). Accurate estimation of cell composition in bulk expression through robust integration of single-cell information. *Nature communications*, 11(1):1971.
3. Lenz, G., Wright, G., Dave, S., Xiao, W., Powell, J., Zhao, H., Xu, W., Tan, B., Goldschmidt, N., Iqbal, J., et al. (2008). Stromal gene signatures in large-b-cell lymphomas. *New England Journal of Medicine*, 359(22):2313–2323.
5. McCarthy, D. J., Chen, Y., and Smyth, G. K. (2012). Differential expression analysis of multifactor rna-seq experiments with respect to biological variation. *Nucleic Acids Research*, 40(10):4288–4297.
18. Newman, A. M., Steen, C. B., Liu, C. L., Gentles, A. J., Chaudhuri, A. A., Scherer, F., Khodadoust, M. S., Esfahani, M. S., Luca, B. A., Steiner, D., et al. (2019). Determining cell type abundance and expression from bulk tissues with digital cytometry. *Nature biotechnology*, 37(7):773–782.
13. Ren, X., Wen, W., Fan, X., Hou, W., Su, B., Cai, P., Li, J., Liu, Y., Tang, F., Zhang, F., et al. (2021). Covid-19 immune features revealed by a large-scale single-cell transcriptome atlas. *Cell*, 184(7):1895–1913.
4. Robinson, M. D., McCarthy, D. J., and Smyth, G. K. (2010). edger: a bioconductor package for differential expression analysis of digital gene expression data. *Bioinformatics*, 26(1):139–140.
9. Roider, T., Seufert, J., Uvarovskii, A., Frauhammer, F., Bordas, M., Abedpour, N., Stolarczyk, M., Mallm, J.-P., Herbst, S. A., Bruch, P.-M., et al. (2020). Dissecting intratumour heterogeneity of nodal b-cell lymphomas at the transcriptional, genetic and drug-response levels. *Nature cell biology*, 22(7):896–906.
15. Saeedi-Boroujeni, A. and Mahmoudian-Sani, M.-R. (2021). Anti-inflammatory potential of quercetin in covid-19 treatment. *Journal of Inflammation*, 18:1–9.
10. Schmitz, R., Wright, G. W., Huang, D. W., Johnson, C. A., Phelan, J. D., Wang, J. Q., Roulland, S., Kasbekar, M., Young, R. M., Shaffer, A. L., et al. (2018). Genetics and pathogenesis of diffuse large b-cell lymphoma. *New England Journal of Medicine*, 378(15):1396–1407.
12. Siska, P. J., Decking, S.-M., Babl, N., Matos, C., Bruss, C., Singer, K., Klitzke, J., Schoen, M., Simeth, J., Koestler, J., et al. (2021). Metabolic imbalance of t cells in covid-19 is hallmarked by basigin and mitigated by dexamethasone. *The Journal of clinical investigation*, 131(22).
8. Steen, C. B., Luca, B. A., Esfahani, M. S., Azizi, A., Sworder, B. J., Nabet, B. Y., Kurtz, D. M., Liu, C. L., Khameneh, F., Advani, R. H., et al. (2021). The landscape of tumor cell states and ecosystems in diffuse large b cell lymphoma. *Cancer cell*, 39(10):1422–1437.
6. Stuart, T., Butler, A., Hoffman, P., Hafemeister, C., Papalexi, E., III, W. M. M., Hao, Y., Stoeckius, M., Smibert, P., and Satija, R. (2019). Comprehensive integration of single-cell data. *Cell*, 177:1888–1902.
17. Wang, J., Roeder, K., and Devlin, B. (2021). Bayesian estimation of cell type–specific gene expression with prior derived from single-cell data. *Genome research*, 31(10):1807–1818.
11. Wang, X., Park, J., Susztak, K., Zhang, N. R., and Li, M. (2019). Bulk tissue cell type deconvolution with multi-subject single-cell expression reference. *Nature Communications*, 10(1).
2. Wright, G., Tan, B., Rosenwald, A., Hurt, E. H., Wiestner, A., and Staudt, L. M. (2003). A gene expression-based method to diagnose clinically distinct subgroups of diffuse large b cell lymphoma. *Proceedings of the National Academy of Sciences*, 100(17):9991–9996.

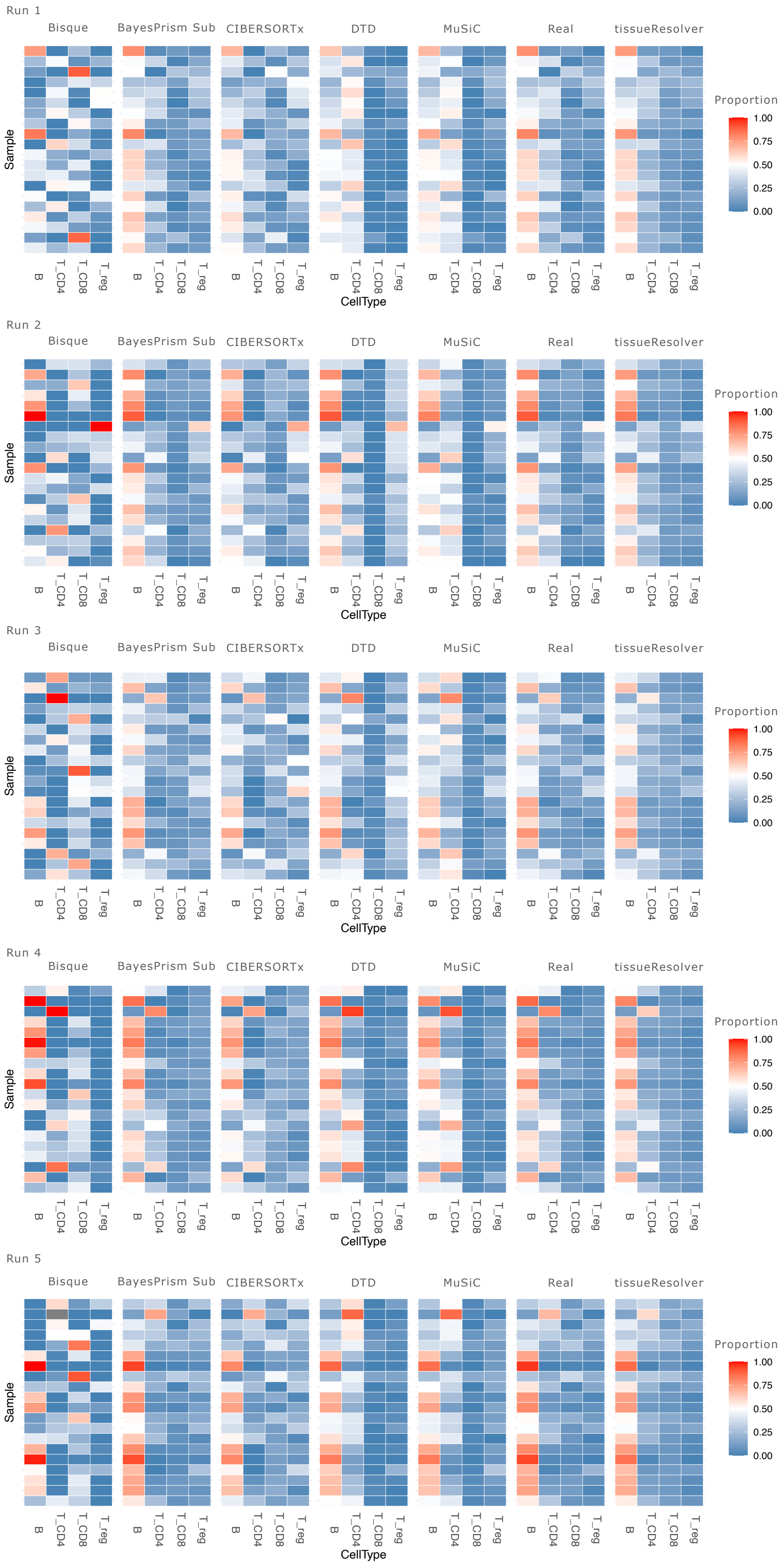

Fig. 15. Explained cell type proportions versus ground truth proportions (real) for all simulation runs for 20 randomly sampled bulks each.

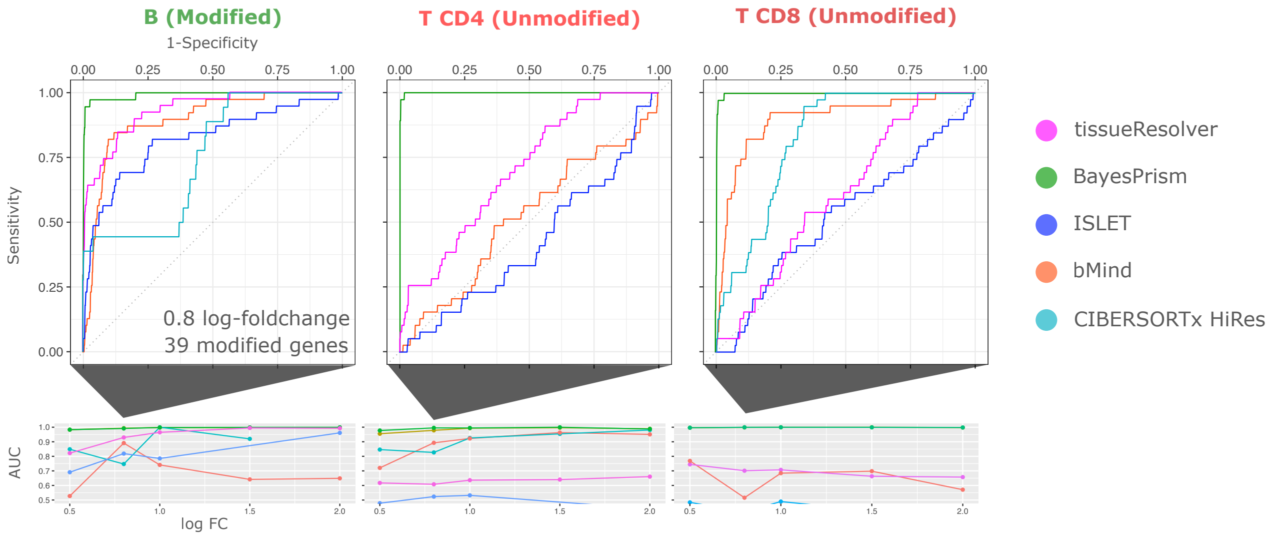

Fig. 16. ROC and AUC curves for the modified B-cells and the (unmodified) CD4 T and B cells. The remaining characteristics are completely analogous to fig. 2. Note that we do only depict BayesPrism with subclustering here, as the non-subclustering version in this case produces almost identical results.

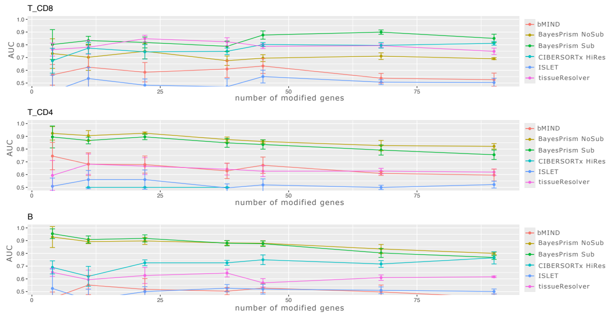

Fig. 17. AUC curves for the modified CD8 T-cells and the (unmodified) CD4 T and B-cells as a function of the number of modified genes. Values significantly larger than 0.5 mean that the modified genes have been attributed to the corresponding cell type, which we expect only for the modified CD8 T-cells, whereas other cell types are expected to give values around 0.5.

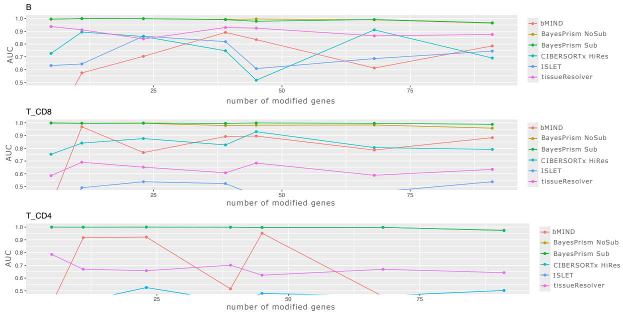

Fig. 18. AUC curves for the modified B-cells and the (unmodified) CD4 T and CD8 T-cells as a function of the number of modified genes. Values significantly larger than 0.5 mean that the modified genes have been attributed to the corresponding cell type, which we expect only for the modified B-cells, whereas other cell types are expected to give values around 0.5.
